# ﻿Four new species of *Marphysa* (Annelida, Eunicida, Eunicidae) from the east coast of Peninsular Malaysia

**DOI:** 10.3897/zookeys.1204.117261

**Published:** 2024-06-04

**Authors:** Che Engku Siti Mariam Che Engku Abdullah, Izwandy Idris, Afiq Durrani Mohd Fahmi, Beth Flaxman, Pat Hutchings

**Affiliations:** 1 Institute of Oceanography and Environment, Universiti Malaysia Terengganu, 21030 Kuala Nerus, Terengganu, Malaysia; 2 South China Sea Repository and Reference Centre, Institute of Oceanography and Environment, Universiti Malaysia Terengganu, 21030 Kuala Nerus, Terengganu, Malaysia; 3 Mangrove Research Unit, Institute of Oceanography and Environment, Universiti Malaysia Terengganu, 21030 Kuala Nerus, Terengganu, Malaysia; 4 Faculty of Science and Marine Environment, Universiti Malaysia Terengganu, 21030 Kuala Nerus, Terengganu, Malaysia; 5 Australian Museum Research Institute, Australian Museum, NSW 2010, Sydney, Australia; 6 School of Life and Environmental Sciences, The University of Sydney, NSW 2006, Sydney, Australia; 7 Marine Ecology Group, School of Natural Sciences, Wallumattagal Campus, Macquarie University, NSW 2109, North Ryde, Australia

**Keywords:** Bloodworm, COI, identification key, mangrove bait worm, *Marphysa*, South China Sea

## Abstract

Four new species of *Marphysa* are described from Terengganu state on the east coast of Peninsular Malaysia, using morphological and molecular (cytochrome oxidase subunit I (COI) gene) data. These species belong to different groups of *Marphysa*: *Marphysakertehensis***sp. nov.** belongs to Group A (Mossambica), *Marphysamerchangensis***sp. nov.** and *Marphysasetiuense***sp. nov.** belong to Group B (Sanguinea) and *Marphysaibaiensis***sp. nov.** belongs to Group E (Gravelyi). *Marphysakertehensis***sp. nov.** is characterised by having only limbate chaetae, absence of subacicular hooks, three types of pectinate chaetae including wide, thick isodont with short and slender inner teeth, and pectinate branchiae with up to nine branchial filaments. *Marphysamerchangensis***sp. nov.** is characterised by the presence of eyes, unidentate subacicular hooks, four types of pectinate chaetae including wide, thick anodont pectinate chaetae with five long and thick inner teeth, and pectinate branchiae with up to six branchial filaments. *Marphysasetiuense***sp. nov.** has mostly unidentate subacicular hooks (bidentate on several posterior chaetigers), four types of pectinate chaetae including wide, thick anodont pectinate chaetae with seven thick and long inner teeth, and pectinate branchiae with up to five branchial filaments. *Marphysaibaiensis***sp. nov.** has bidentate subacicular hooks throughout, five types of pectinate chaetae, including a heterodont with 12 short and slender inner teeth, and pectinate branchiae with up to eight branchial filaments. The designation of these new species based on morphology is fully supported by molecular data. Habitat descriptions of each species are also included.

## ﻿Introduction

*Marphysa* de Quatrefages 1866, currently with 83 accepted species, is the second most speciose genus in the family Eunicidae, after the genus *Eunice* Cuvier, 1817 (Read and Fauchald 2023). *Marphysa* species inhabit a wide range of habitats, either in soft sediments or rocky ground, from intertidal to shallow subtidal depth, and are commonly found in estuarine or sheltered habitats (Abdullah et al. in review; Zanol et al. 2016; Martin et al. 2020). Three species have recently been described from the deep sea (Lavesque et al. 2023). Taxonomic studies of *Marphysa* species have increased considerably since the redescription of the type species, *M.sanguinea* (Montagu, 1813) and the designation of a neotype by Hutchings and Karageorgopoulos (2003); and later by molecular sequencing (Zanol et al. 2010). Since then, many more species have been described or previously synonymised species resurrected, using molecular data and additional morphological characters such as the types and distributions of chaetae.

According to Fauchald (1970) and Glasby and Hutchings (2010), *Marphysa* can be divided into five informal groups (Groups A–E) depending on their type of chaetae. Group A (Mossambica) without compound chaetae, Group B (Sanguinea) with only compound spinigers present, Group C (Aenea) with only compound falcigers present, Group D (Belli) with both spinigers and falcigers present, and Group E (Teretiuscula; renamed Gravelyi by Molina-Acevedo and Idris 2021) with only compound spinigers and subacicular chaetae in anterior parapodia and limbate chaetae throughout.

In Malaysia, Paxton and Chou (2000) suggested that the low number of polychaete species identified in Malaysia is an underestimate due to the limited sampling of polychaetes. Idris et al. (2014) described *Marphysamoribidii* Idris, Hutchings & Arshad, 2014 from the west coast of Peninsular Malaysia. The species is currently the only *Marphysa* described from Malaysia, occurring in *Rhizophora* and *Sonneratia* spp. mangroves. *Marphysamoribidii* is regularly used as fishing bait by local fishermen. In addition, recent studies (Ee Pei et al. 2020; Rapi et al. 2020; Rosman et al. 2020) reported the potential applications of *M.moribidii* as a wound-healing agent and bio-catalyst of gold and silver nanoparticles. This study investigated *Marphysa* species from the mangrove forest on the east coast of Peninsular Malaysia, specifically Terengganu, as they may also have potential applications similar to *M.moribidii*. We found four new *Marphysa* species using an integrated approach to taxonomy, including morphological and molecular analyses.

## ﻿Materials and methods

### ﻿Study area and sampling

**Figure 1.**
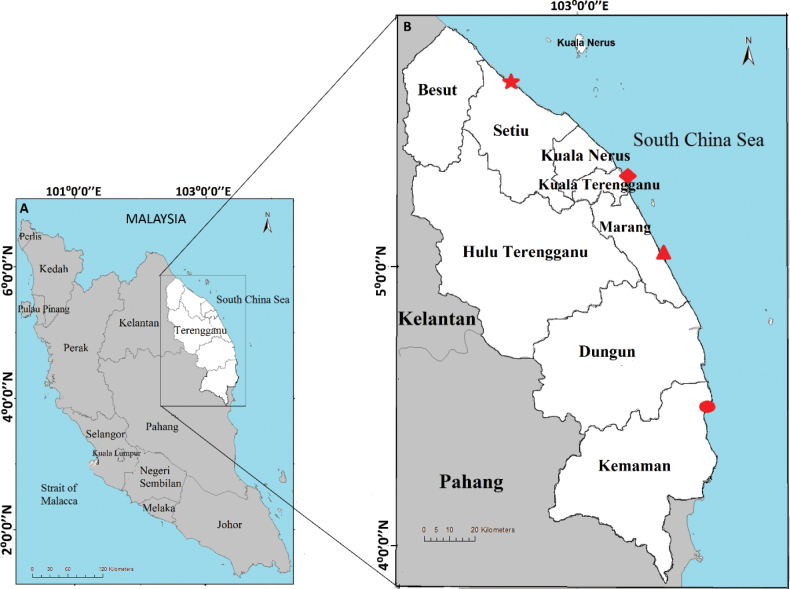
Map showing sampling sites of four new *Marphysa* species in Terengganu mangrove forest, east coast of Peninsular Malaysia **A** location of Terengganu on the east of Peninsular Malaysia **B** symbols indicate each sampling site; Setiu Wetlands (red star), Kuala Ibai (red rhombus), Merchang (red triangle), and Kerteh (red oval).

*Marphysa* specimens were collected from the rivers, lagoon and estuary of the Terengganu mangrove forests during spring low tides from September 2021 until March 2022. A total of four mangrove areas were chosen, i.e. Setiu wetlands, Kuala Ibai, Merchang, and Kerteh (Fig. 1A, B). At each site, sediments were dug using a shovel to approximately 30 cm depth at several points along the river (upper course to lower course) and carefully broken into small pieces to search for the worms. Worms suspected to be *Marphysa* were fixed and preserved in 95% ethanol. Sediments where the *Marphysa* worms were found were also collected and kept in labelled plastic bags for sediment analysis. All material was collected by the first author.

### ﻿Morphological analyses

**Figure 2.**
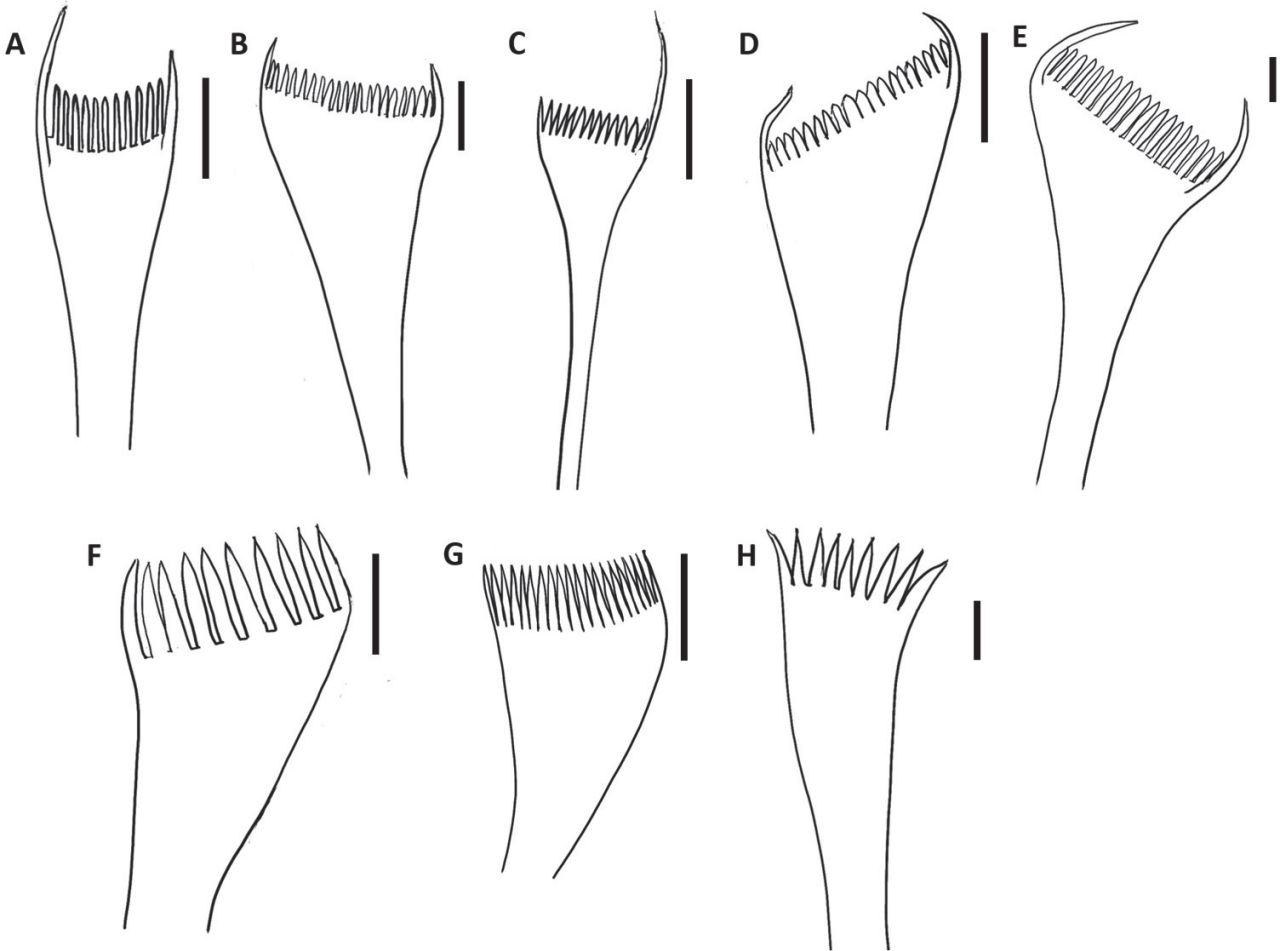
The schematic drawing of type of pectinate chaetae present in *Marphysa* from Terengganu **A** thin, narrow isodont with short and slender inner teeth (type 1) **B** thin, wide isodont with short and slender inner teeth (type 2) **C** thin, narrow heterodont with short and slender inner teeth (type 3) **D** thick, wide isodont with short and slender inner teeth (type 4) **E** thick, wide isodont with short and slender inner teeth (type 5) **F** thick, narrow anodont with long and thick inner teeth (type 6) **G** thick, wide anodont with long and slender inner teeth (type 7) **H** thick, wide anodont with long and thick inner teeth (type 8). Scale bars: 18 µm (**A**); 35 µm (**B, G**); 20 µm (**C**); 38 µm (**D**); 32 µm (**E**); 13 µm (**F**); 30 µm (**H**).

Preserved specimens were examined under AmScope SM-2 Series stereo and 120 Series compound microscopes. Additionally, the specimens were also examined under Leica M165 C stereo and Nikon Labophot-2 compound microscopes, and photographed with a Nikon D610 camera at the Natural History Museum of Los Angeles County, USA (NHMLAC). Drawings of parapodia and pectinate chaetae were made using a Wacom Intuos Pro drawing tablet. Length at chaetiger 10 (L10) and width at chaetiger 10 (W10) without parapodia of all specimens were measured and recorded. Morphological terminology, including diagnostic features of *Marphysa* species, follows Molina-Acevedo and Carrera-Parra (2017). Terminology of pectinate chaetae is derived and modified from Carrera-Parra and Salazar-Vallejo (1998) for the relative length of outer and inner teeth, Zanol et al. (2014, 2016) for the thickness of the blade and Glasby et al. (2019) for the size of the inner teeth: isodont means outer teeth much longer than inner teeth; anodont means outer teeth more or less the same length as inner teeth; heterodont refers to when one long and one short (same length as inner teeth) lateral tooth is present. The thickness of the shaft is thin when it is thinner than the limbate chaetae on the same parapodium, thick when the shaft is as thick or thicker than limbate chaetae on the same parapodium. The width of the pectinate blade is wide when the blade is ≥ 30 μm and narrow below this threshold; length of the inner teeth is long when they are ≥ 12 µm and thick when ≥ 2 µm; below these thresholds, the teeth are defined as short and slender, respectively. Table 1 and Fig. 2 summarises and illustrates the types of pectinate chaetae present in species of *Marphysa* from Terengganu.

**Table 1.** Type of pectinate chaetae present in *Marphysa* from Terengganu.

Type of pectinate chaetae	Description
**Type 1**	Thin, narrow isodont with short and slender inner teeth
**Type 2**	Thin, wide isodont with short and slender inner teeth
**Type 3**	Thin, narrow heterodont with short and slender inner teeth
**Type 4**	Thick, wide isodont with short and slender inner teeth
**Type 5**	Thick, wide isodont with long and slender inner teeth
**Type 6**	Thick, narrow anodont with long and thick inner teeth
**Type 7**	Thick, wide anodont with long and slender inner teeth
**Type 8**	Thick, wide anodont with long and thick inner teeth

Terminology of maxillary apparatus followed Molina-Acevedo and Carrera-Parra (2015). Several parapodia from the anterior, median, and posterior regions were removed from the type material of each species, dehydrated in ethanol and hexamethyldisilazane (HMDS), coated with 20 nm of silver-gold, examined under the scanning electron microscope JEOL JSM-6360LA, and imaged with a secondary detector at SEM laboratories of Universiti Malaysia Terengganu and Macquarie University, Sydney, Australia.

### ﻿Repositories

Materials were deposited at the institution and museums listed below:

- South China Sea Repository and Reference Centre (**RRC**), Universiti Malaysia Terengganu, Malaysia, as holotype (**UMT**) (UMTAnn 2149, UMTAnn 2177, UMTAnn 2179, UMTAnn 2181) and paratypes (UMTAnn 2150 to 2176, UMTAnn 2178, UMTAnn 2180, UMTAnn 2182 to 2193)
- Australian Museum, Sydney, Australia (**AM**) as paratypes (AM W.54041 to W.54060)
- Natural History Museum of Los Angeles County, USA (**NHMLAC**) as paratypes (LACM-AHF 13494 to 13505)
- Lee Kong Chian Natural History Museum, Singapore (**LKCNHM**) as paratypes (ZRC.ANN.1604 to ZRC.ANN.1612, ZRC.ANN.1614 to 1615)
- Iziko South African Museum, South Africa (**ISAM**) as paratypes (SAM-MB-A096021 to A096023)

### ﻿Molecular analyses

Molecular analyses were done at the Universiti Malaysia Terengganu (UMT) and the Australian Museum Research Institute, Australian Museum, Sydney (AMRI). At UMT, extractions of DNA were done using the xanthogenate method (Tillett and Neilan 2000). Approximately 600 bp of cytochrome oxidase subunit 1 (COI) gene were amplified using universal primer pair LCO1490 and HCO2198 (Folmer et al. 1994). Polymerase Chain Reaction (PCR) amplifications were carried out using 12.5 μL of OneTaq Quick-Load Master mix, 9.5 μL of biology grade water, 0.5 μL of primers (10 μM), 1 μL of 1% bovine serum albumin (BSA) and 1 μL DNA template. The temperature profile was as follows: 95 °C / 180 s – (94 °C / 20 s – 45 °C / 30 s – 72 °C / 60 s)*35 cycles and final extension time at 72 °C / 300 s. PCR success was verified by electrophoresis in a 1% p/v agarose gel stained with GelRed. Amplified products were sent to Apical Scientific Sdn. Bhd. for Sanger sequencing using forward primer (LCO1490).

Meanwhile, at AMRI, extractions of DNA were done with an ISOLATE II Genomic DNA kit (BIOLINE) following the protocol supplied by the manufacturers. Approximately 600 bp of COI gene were amplified using primers polyLCO and polyHCO (Carr et al. 2011). PCR was performed with Taq DNA Polymerase QIAGEN Kit in 20 μL mixtures containing: 2 μL of 10× CoralLoad PCR Buffer (final concentration of 1×), 1.5 μL of MgCl_2_ (25 Mm) solution, 1.5 μL of PCR nucleotide mix (final concentration of 0.2 mM each dNTP), 0.4 μl of each primer (final concentration of 0.2 μM), 0.1 μl of Taq DNA Polymerase (5U/μl), 1 μl template DNA and 13.1 μL of nuclease-free water. The temperature profile was as follows; 94 °C / 60 s – (94 °C / 40 s – 45 °C / 40 s – 72 °C / 60 s)*5 cycles – (94 °C / 40 s – 51 °C/ 40 s – 72 °C / 60 s)*35 cycles – 72 °C / 300 s. PCR success was verified by electrophoresis in a 1% p/v agarose gel stained with GelRed. Amplified products were sent to Macrogen Company for Sanger sequencing using the same set of primers used for PCR.

A total of 63 COI sequences were downloaded from GenBank or obtained during this study; 60 COI sequences of *Marphysa* species and three outgroup species from closely related genera in the order Eunicida (Table 2). All COI sequences were aligned in MEGA v. 11.0.10 using ClustalW plugin with default settings. The best DNA/Protein Models (ML) test was conducted, and the GTR model of molecular evolution was chosen as the best evolutionary model for the COI gene alignment. The phylogenetic analysis was performed in MEGA v. 11.0.10 (Tamura et al. 2021). The analysis was run for 1000 replicates. Pair-wise Kimura 2-parameter (K2P) genetic distance was performed using MEGA v. 11.0.10.

**Table 2.** Terminal taxa used in molecular part of the study (COI), with type localities, collection localities, GenBank accession numbers and references.

Species	Type locality	Collection locality	GenBank accession number	Reference
*Ophryotrochamarinae* Zhang, Zhou, Yen, Hiley & Rouse, 2023	Gulf of California, Mexico	Hydrothermal vents of Pescadero and Guaymas Basin, Gulf of California, Mexico	OP561817	Zhang et al. (2023)
*Diopatraaciculata* Knox & Cameron, 1971	Port Phillip Bay, Victoria, Australia	Port Phillip Bay, Victoria, Australia	AY838867	Struck et al. (2006)
*Oenonefulgida* (Lamarck, 1818)	Coast of Red Sea, Egypt	Coast of Red Sea, Egypt	AY838872	Struck et al. (2006)
*Marphysaaegypti* Elgetany, El-Ghobashy, Ghoneim & Struck, 2018	Suez Canal, Egypt	Suez Canal, Egypt	MF196968	Elgetany et al. (2018)
*Marphysabifurcata* Kott, 1951	Western Australia, Australia	Queensland, Australia	KX172177	Zanol et al. (2016)
			KX172178	
*Marphysabrevitentaculata* Treadwell, 1921	Tobago Island, Trinidad and Tobago	Quintana Roo, Mexico	GQ497548	Zanol et al. (2010)
*Marphysacalifornica* Moore, 1909	California, USA	California, USA	GQ497552	Zanol et al. (2010)
*Marphysachirigota* Martin, Gil & Zanol, 2020	Bay of Cadiz, Spain	Bay of Cadiz, Spain	MN816443	Martin et al. (2020)
*Marphysadavidattenboroughi* Lavesque, Zanol, Daffe, Flaxman & Hutchings, 2023	Bass Strait, Australia	Bass Strait, Australia	OQ622195	Lavesque et al. (2023)
			OQ622196	
*Marphysadisjuncta* Hartman, 1961	California, USA	California, USA	GQ497549	Zanol et al. (2010)
*Marphysafauchaldi* Glasby & Hutchings, 2010	Northern Territory, Australia	Northern Territory, Australia	KX172165	Zanol et al. (2016)
*Marphysagaditana* Martin, Gil & Zanol, 2020	Bay of Cadiz, Spain	Bay of Cadiz, Spain	MN816441	Martin et al. (2020)
*Marphysahongkongensa* Wang, Zhang & Qiu, 2018	Hong Kong	Hong Kong	MH598525	Wang et al. (2018)
			MH598526	
*Marphysaibaiensis* sp. nov.	Kuala Ibai, Terengganu, Malaysia	Kuala Ibai Lagoon and estuary, Terengganu, Malaysia	OR995540	This study
			OR995541	
			OR995542	
			OR995543	
			OR995544	
			OR995545	
*Marphysailoiloensis* Glasby, Mandario, Burghardt, Kupriyanova, Gunton & Hutchings, 2019	Iloilo, Philippines	Iloilo, Philippines	MN106279	Glasby et al. (2019)
			MN106280	
*Marphysakertehensis* sp. nov.	Kerteh, Terengganu, Malaysia	Kerteh mangrove river, Terengganu, Malaysia	OR981603	This study
			OR981604	
			OR981605	
			OR995527	
			OR995528	
			OR995529	
			OR995530	
			OR995531	
*Marphysakristiani* Zanol, da Silva & Hutchings, 2016	New South Wales, Australia	New South Wales, Australia	KX172160	Zanol et al. (2016)
			KX172161	
*Marphysamadrasi* Hutchings, Lavesque, Priscilla, Daffe, Malathi & Glasby, 2020	Chennai, India	Chennai, India	MT813506	Hutchings et al. (2020)
			MT813507	
*Marphysamerchangensis* sp. nov.	Merchang, Terengganu, Malaysia	Merchang mangrove estuary, Terengganu, Malaysia	OR995532	This study
			OR995533	
			OR995534	
			OR995535	
*Marphysamossambica* (Peters, 1854)	Mozambique	Iloilo, Philippines	KX172164	Zanol et al. (2016)
*Marphysamullawa* Hutchings & Karageorgopoulus, 2003	Queensland, Australia	New South Wales, Australia	KX172166	Zanol et al. (2016)
			KX172167	
*Marphysapapuaensis* Lavesque, Daffe, Glasby, Hourdez & Hutchings, 2022	Solomon Sea, Papua New Guinea	Solomon Sea, Papua New Guinea	OP184050	Lavesque et al. (2022)
*Marphysapseudosessiloa* Zanol, da Silva & Hutchings, 2017	New South Wales, Australia	New South Wales, Australia	KY605405	Zanol et al. (2010)
			KY605406	
*Marphysaregalis* Verill, 1900	Bermuda, British Overseas Territory	Ceara, Brazil (Lavesque et al. 2023)	GQ497562	Zanol et al. (2010)
*Marphysasanguinea* (Montagu, 1813)	Devon, UK	Callot Island, France	GQ497547	Zanol et al. (2010)
*Marphysasanguinea* (Montagu, 1813)	Devon, UK	Cornwall, UK	MK950853	Lavesque et al. (2019)
*Marphysasanguinea* (Montagu, 1813)	Arcachon Bay, France	Arcachon Bay, France	MK541904	Lavesque et al. (2019)
*Marphysasetiuense* sp. nov.	Setiu Wetlands, Terengganu, Malaysia	Setiu Wetland estuary, Terengganu Malaysia	OR995536	This study
			OR995537	
			OR995538	
			OR995539	
*Marphysasherlockae* Kara, Molina-Acevedo, Zanol, Simon & Idris, 2020	Durban, South Africa	Strand, South Africa	MT840349	Kara et al. (2020)
			MT840350	
*Marphysatripectinata* Liu, Hutchings & Sun, 2017	Beihai, China	Beihai, China	MN106271	Liu et al. (2017)
			MN106272	
*Marphysavictori* Lavesque, Daffe, Bonifácio & Hutchings, 2017	Arcachon Bay, France	Arcachon Bay, France	MG384996	Lavesque et al. (2017)
*Marphysavictori* Lavesque, Daffe, Bonifácio & Hutchings, 2017	Mangoku-ura Inlet, Japan	Mangoku-ura Inlet, Japan	LC467767	Abe et al. (2019)
*Marphysavictori* Lavesque, Daffe, Bonifácio & Hutchings, 2017	Arcachon, France	Ena Bay, Japan	LC467772	Abe et al. (2019)
*Marphysaviridis* Treadwell, 1917	Florida, USA	Ceara, Brazil	GQ497553	Zanol et al. (2010)
*Marphysazanolae* Lavesque, Daffe, Glasby, Hourdez & Hutchings, 2022	Solomon Sea, Papua New Guinea	Solomon Sea, Papua New Guinea	OP184049	Lavesque et al. (2023)

### ﻿Habitat description and sediment analyses

Habitats of identified *Marphysa* were described based on the observations made during sampling including mangrove vegetations and sediment analyses. The particle size of the sediments was determined using dry-sieving techniques. Sediments were oven-dried at 60 °C for ~ 72 h. Then, 100 g of sub-samples were gently dry-sieved through a series of 4, 2, 1, 0.5, 0.25, 0.125, and 0.063 mm mesh openings of an Octagon D200 Digital mechanical shaker. Sediments retained on each sieve were weighed and recorded. Sediment grain size was classified according to grain size classifications by Blair and McPherson (1999), modified after Udden (1914) and Wentworth (1922). The percentage of particle size compositions was calculated, and the texture of sediments was determined based on the sediment textural classification scheme of Blair and McPherson (1999), modified after Folk et al. (1970).

Furthermore, total organic matter was determined using the loss on ignition (LOI) method which calculates the weight loss after combustion (Dean 1974). A total of 5 g of oven-dried sediments were placed in ceramic crucibles and ashed at 550 °C for six h in a muffle furnace. Then, sediments were cooled in a desiccator and weighed. The percentage of total organic matter (TOM) was analysed by the percentage loss of weight on ignition at 550 °C.

## ﻿Results

### ﻿Molecular analyses

**Figure 3.**
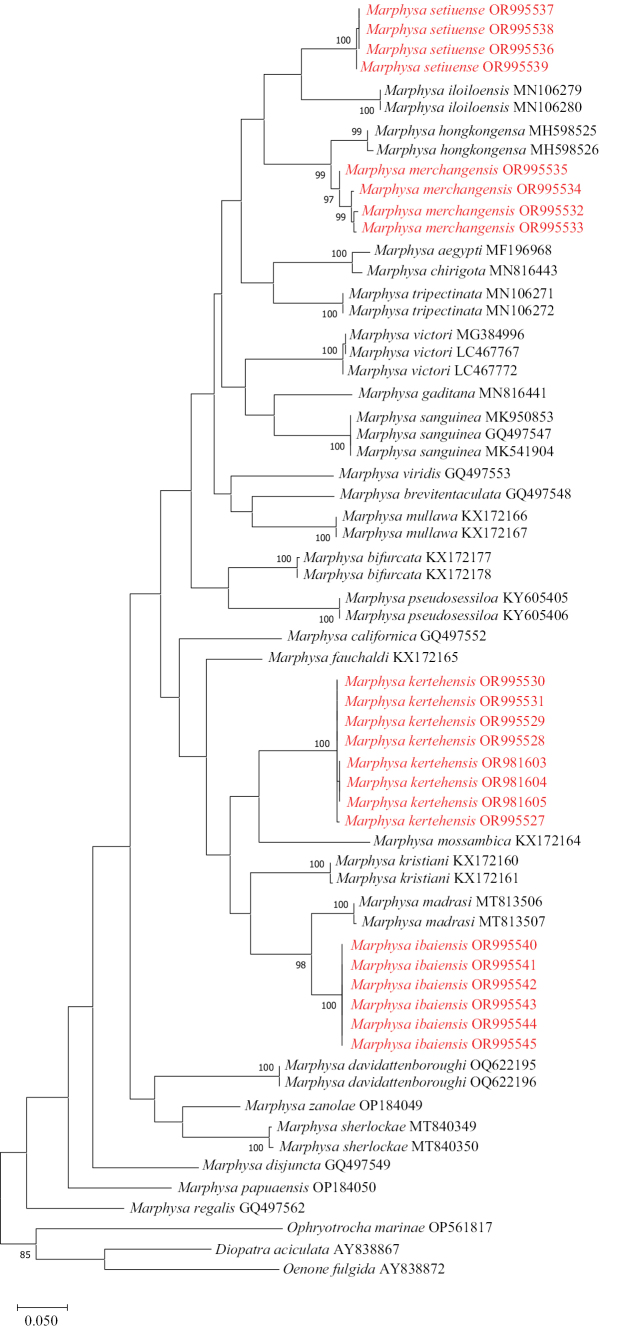
Phylogenetic tree generated by maximum likelihood (ML) method based on COI (460 bp). The sequences of the four new species of *Marphysa* obtained in this study are marked in red. Numbers beside the branches indicate ML bootstrap values of 80 (maximum: 100) based on 1000 bootstrap replications.

DNA sequences of COI (460 bp) (Fig. 3) were used for phylogenetic analysis based on the maximum likelihood (ML) method. Results based on the COI showed that the four *Marphysa* species from Terengganu were well separated from other sequences of *Marphysa* and formed four different clades. Nodal support ranges from 97–100%, showing strong support for the clades. The interspecific divergence between these new species and all their sister taxa pair is high (pair-wise Kimura 2-parameter – COIK2P range from 6.14%–19.16%) (see Suppl. material 1).

### ﻿Ecological analyses

Particle size analyses of sediment from sampling sites in Terengganu mangrove forest estuary, lagoon and river are shown in Table 3. Generally, sediments from all sampling sites were mainly composed of sand. However, sites can be differentiated by the composition of different particle sizes, sediment texture, and percentage of organic matter content. Sediments collected from Setiu Wetlands, Kuala Ibai mangrove estuary, and Kerteh mangrove river were dominated by fine sand; meanwhile, sediments from Merchang mangrove estuary and Kuala Ibai lagoon were dominated by a mixture of fine pebble + granule and medium size sand, respectively.

**Table 3.** Particle size composition (%) of sediments from four sampling sites in Terengganu mangrove forest. Asterisk (*) indicates the largest particle size composition.

Particle size composition (%)					
	Setiu Wetlands	Merchang mangrove estuary	Kuala Ibai		Kerteh mangrove river
			Mangrove estuary	Lagoon	
Fine pebble + granule (gravel)	3.06	*24.59	2.9	3.94	7.99
Very coarse sand	8.64	19.77	4.34	12.49	9.4
Coarse sand	15.21	21.33	7.59	23.89	8.24
Medium sand	26.29	17.6	21.28	*46.03	12.78
Fine sand	*32.08	7.05	*49.92	13.2	*33.16
Very fine sand	11.51	7.38	12.55	0.45	20.89
Silt + clay (mud)	3.21	2.28	1.42	0	7.54
**Total**	100	100	100	100	100
**Percentage sand**	93.73	73.13	95.68	96.06	84.47

**Figure 4.**
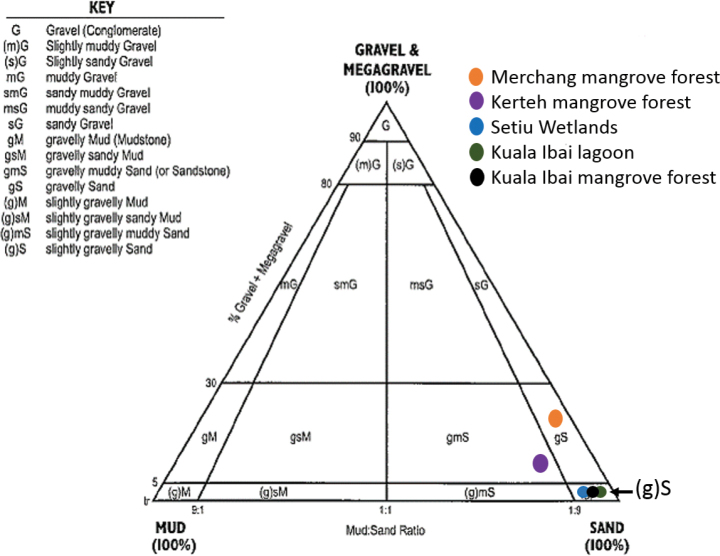
Sediment classification from sampling sites of Terengganu mangrove forest, according to sediment classification scheme by Blair and McPherson (1999), modified after Folk et al. (1970).

All sampling sites were located less than 1 km from the river mouth except for Kerteh station, which is 3.12 km from the river mouth. The sediment texture of sampling sites in Terengganu mangrove forest was classified as slightly gravelly sand, gravelly sand, and gravelly muddy sand (Fig. 4, Table 4). Total organic matter content indicated in Table 4; ranges from 0.29 ± 0.05%–5.11 ± 0.91%.

**Table 4.** Distance of sampling sites from river mouth, type of sediment textures and total organic matter content of sampling sites in Terengganu mangrove forest.

Sampling sites	Distance from river mouth (km)	Type of sediment texture	Organic matter content (%)
Setiu Wetlands	0.9	Slightly gravelly sand	1.02 ± 0.17
Merchang mangrove estuary	0.85	Gravelly sand	5.11 ± 0.91
Kuala Ibai mangrove estuary	0.83	Slightly gravelly sand	1.66 ± 0.89
Kuala Ibai lagoon	0.53	Slightly gravelly sand	1.97 ± 0.29
Kerteh mangrove river	3.12	Gravelly muddy sand	0.29 ± 0.05

### ﻿Taxonomic account

**Family Eunicidae Berthold, 1827**

**Order Eunicida Dales, 1962**

#### ﻿Genus Marphysa

Taxon classificationAnimaliaEunicidaEunicidae

Quatrefages, 1866

CE19ECA5-D74E-5865-9139-30C34E6E3278

##### Type species.

*Nereissanguinea* Montagu, 1813.

##### Diagnosis

**(after Molina-Acevedo and Carrera-Parra 2017).** Prostomium slightly or completely bilobed; five prostomial appendages without articulations; eyes present or absent. Peristomium without peristomial cirri. Maxillary apparatus with four pairs of maxillae, an unpaired on the left side; MI with falcal arch developed, extended, with the outer edge of the base arched; MIII curved, forming part of distal arc, with attachment lamella of rectangular or irregular shape, situated at centre of posterior edge of maxilla; MIV with circular or rectangular attachment lamella. Branchiae distributed along entire body. Dorsal cirri without articulation; postchaetal lobe well developed in anterior region. Ventral cirri with swollen, oval, or circular base. All sub-aciculae dark. Supracicular chaetae include limbate, pectinate isodont chaetae with slender teeth, pectinate anodont chaetae with long teeth. Subacicular chaetae include compound falcigers or spinigers, or only limbate chaetae. Subacicular hook dark or translucent, bidentate or unidentate. Pygidium with two pairs of anal cirri, without articulation.

#### ﻿ Marphysa kertehensis sp. nov.

Taxon classificationAnimaliaEunicidaEunicidae

BF9FE410-DC1E-56E4-9E08-65806C6310C6

https://zoobank.org/73FBB175-0342-4A83-AF27-36A1DCD5EB66

**Figure 5.**
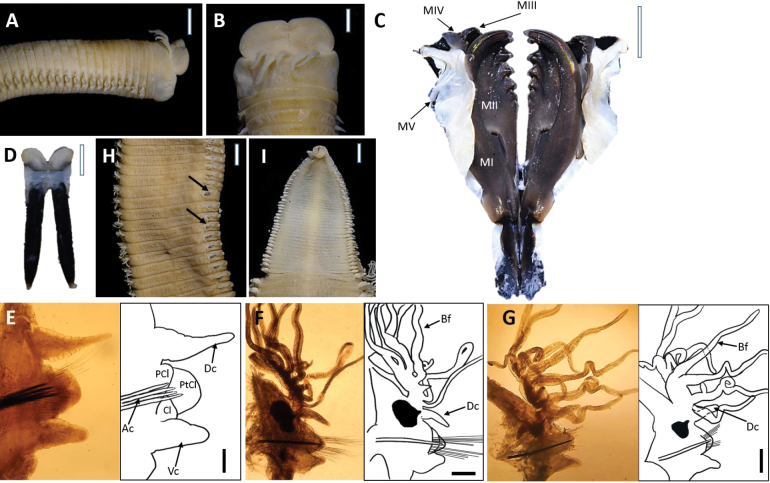
*Marphysakertehensis* sp. nov. Holotype UMTAnn 2181 (**A–I**). Light microscopy images and digital drawing **A** anterior end, lateral view **B** anterior end, dorsal view **C** maxillae, dorsal view **D** mandibles, dorsal view **E** parapodium, chaetiger 10 **F** parapodium, chaetiger 295 **G** parapodium, chaetiger 462 **H** median region, dorsal view. Arrows indicate black dot at the base of dorsal cirri **I** posterior segments and pygidium, ventral view. Abbreviations; MI–MV: maxillae I–V, Ac: aciculae, Dc: dorsal cirrus, Vc: ventral cirrus, PCl: prechaetal lobe, Cl: chaetal lobe, PtCl: postchaetal lobe, Bf: branchial filament. Scale bars: 2 mm (**A, H, I**); 1 mm (**B–D**); 0.1 mm (**E–G**).

**Figure 6.**
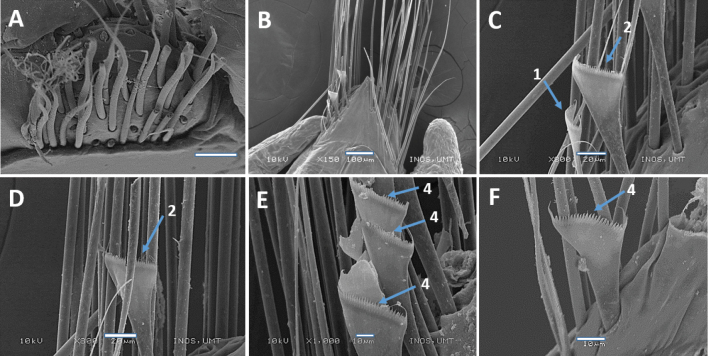
Scanning Electron Microscopy (SEM) images of *Marphysakertehensis* sp. nov. Holotype UMTAnn 2181 (**B–E**), paratype AM W.54059 (**A, F**) **A** limbate chaetae, chaetiger 10 **B** parapodia, chaetiger 300 **C–D** pectinate chaetae, chaetiger 300 **E** pectinate chaetae, chaetiger 462 **F** pectinate chaetae, chaetiger 525. Numbers denoted by arrows indicate the type of pectinate chaetae: 1. Thin, narrow isodont; 2. Thin, wide isodont; 4. Thick, wide isodont. Scale bars: 50 µm (**A**); 100 µm (**B**); 20 µm (**C–D**); 10 µm (**E–F**).

**Figure 7.**
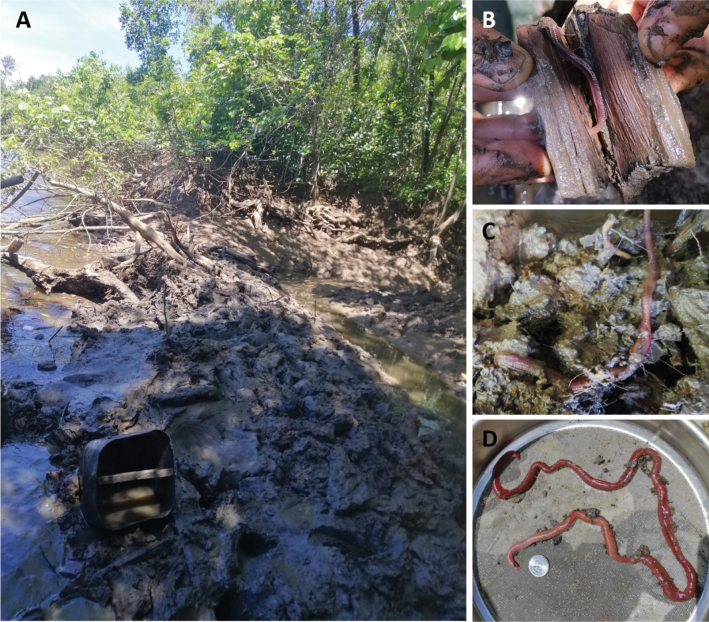
Sampling site in Kerteh mangrove forest (river area) **A** habitat of *Marphysakertehensis* sp. nov. **B***Marphysakertehensis* sp. nov. found inside driftwood and **C** in the sediment **D** live and complete *M.kertehensis* sp. nov.

Figs 1 , 2 , 5 , 6 , 7

##### Material examined.

***Holotype*.** UMTAnn 2181, complete (regenerated posterior), antero-ventrally dissected, some parapodia removed and mounted for SEM. ***Paratypes*.**AM W.54059, complete, some parapodia removed and mounted for SEM; LACM-AHF 13503 to 13505, complete, some parapodia removed; ZRC.ANN.1614 to 1615, incomplete, some parapodia removed; SAM-MB-A096023, incomplete, some parapodia removed. All material was collected from the east coast of Peninsular Malaysia, Terengganu, Kerteh mangrove forest river (04°32.142'N, 103°26.363'E), March 2022.

##### Diagnosis.

Prostomium completely bilobed, five prostomial appendages without articulations; eyes absent. Peristomium without peristomial cirri. Maxillary apparatus with four pairs of maxillae, an unpaired one on the left side, MI with falcal arch extended at sub-right angle, basal outer edge arched, basal inner edge lacking curvature. MII with triangular teeth and without attachment lamella. MIII slightly curved, with equal-sized triangular teeth, without attachment lamella. MIV with dark and curved attachment lamella. Branchiae distributed along entire body. Dorsal cirri without articulations; postchaetal lobe well developed in anterior regions. Ventral cirri with swollen, inflated base. Sub-aciculae black, blunt and translucent at distal end, pale brown in posterior-most parapodia. Supra-acicular chaetae include limbate, pectinate thin, narrow and wide isodont with short and slender inner teeth, and pectinate thick, wide isodont with short and slender inner teeth. Subacicular chaetae include only limbate chaetae. Subacicular hook absent. Pygidium with two pairs of anal cirri, without articulation.

##### Description

**(based on holotype, with variation in parentheses for paratypes).** Preserved specimens beige (Fig. 5A), with 518 (135–578) chaetigers, ~ 413 mm (173–295) total length, 12 mm (6–10.8) in length to chaetiger 10 (L10), 4.8 mm (3.15–5.1 mm) width at chaetiger 10 (W10), excluding parapodia. Body with dorsum convex and flat ventrum (Fig. 5A), without groove; body elongated, rounded in cross-section at anterior and median regions, and dorsoventrally flattened thereafter. Live specimens red (Fig. 7D).

Prostomium bilobed, anteriorly rounded with two dorsoventrally flattened lobes separated by an anterior notch between (Fig. 5B). Prostomial appendages in a semicircle, median antennae separated by a gap (Fig. 5B). Palps reach to first ring of peristomium; lateral and median antennae to second ring of peristomium. Palpophores and ceratophores are ring-shaped, short, thin; palpostyles and ceratostyles tapering and slender. Prostomial peduncles absent. Peristomium larger and wider than prostomium; first ring 3× longer than second ring, separation between rings distinct on all sides.

Maxillae dark (Fig. 5C), and maxillary formula (MF) as follows: 1+1, 5+5 (4–5), 8 (7–8)+0, 3 (3–4)+9 (8–9), 1+1. Maxillary carrier ~ 2.8× shorter than MI, rectangular anteriorly, triangular posteriorly. MI forceps-like, without attachment lamellae, falcal arch extended at sub-right angle, basal outer edge arched, basal inner edge lacking a curvature. Closing system ~ 4.2× shorter than MI. Ligament between MI and MII dark. MII without attachment lamella, teeth triangular, distributed on < 1/2 of plate length. Ligament between MII and MIII dark. MIII single, longer than left MIV, slightly curved, with equal-sized triangular teeth, without attachment lamella. Left MIV short (< 1/2 the size of right MIV), attachment lamella dark, curved. Right MIV long, with teeth triangular, decreasing in size and teeth curved posteriorly; attachment lamella curved, dark. MV paired, longer than high. Mandible dark, longer than MI; cutting plates whitish (Fig. 5D).

First and second parapodia located ventrolaterally but gradually positioned dorsolaterally on subsequent segments. Chaetal lobes conical and directed to ventral cirri in anterior chaetigers, conical in median and posterior chaetigers (Fig. 5E–G). Prechaetal lobe shorter than chaetal lobe throughout body. Postchaetal lobe rounded and longer than chaetal lobe in anterior chaetigers, conical in mid-body onwards and absent in the posterior-most chaetigers. Dorsal cirri digitiform with slender and tapering tips longer than ventral cirri anteriorly, digitiform and slightly longer from mid-body, digitiform and approximately similar length in posterior-most chaetigers (Fig. 5E–G). Ventral cirri digitiform in first chaetigers, basally inflated with digitiform tip from chaetiger 15 onwards (Fig. 5E–G). Branchiae pectinate, from chaetiger 41 (27–58), branchial filaments 3× longer than dorsal cirri where best developed; number of filaments increasing from five anteriorly to nine in mid-body, decreasing to three in last several chaetigers. Black dot present at the base of dorsal cirri from median chaetigers toward posterior chaetigers (Fig. 5F–H).

Notoaciculae absent, neuroaciculae black, blunt, and translucent at distal end along most of body, pale brown in posterior-most parapodia; three or four per parapodium in anterior, one or two per parapodium in median and posterior chaetigers (Fig. 5E–G). Supra-acicular chaetae with limbate capillaries and pectinates, subacicular chaetae with limbate capillaries, compound chaetae absent (Fig. 6A, B). Three types of pectinate chaetae were identified (types 1, 2, 4; see Fig. 2): type 1: thin, narrow isodont with 28 short and slender inner teeth, outer teeth longer on one side, present only in the anterior body region (Fig. 6C); type 2: thin, wide isodont with ~ 30–32 short and slender teeth, present only in median and posterior region (Fig. 6C, D); type 4: thick, wide isodont with ~ 23 short and slender inner teeth, present only in posterior region (Fig. 6E, F). Anodont pectinate chaetae and subacicular hooks (*n* = 30) completely absent. Pygidium with crenulated margin, with two pairs of pygidial cirri attached (Fig. 5I).

##### Etymology.

The new name denotes the type locality (Kerteh River) where the specimens were collected.

##### Type locality.

South China Sea, Malaysia, east coast of Peninsular, Terengganu, Kerteh River (see Fig. 1).

##### Distribution.

Known only from the type locality.

##### Habitat.

Gravelly muddy sand (Table 4), burrowing inside driftwood, in mangroves, intertidal (Fig. 7A–C) with salinity 3.18‰ during spring low tide.

##### Remarks.

With the presence of only limbate chaetae in both supra- and subacicular chaetae bundles, *Marphysakertehensis* sp. nov. belongs to *Marphysa* Group A (Mossambica). Comparing *Marphysa* Group A from Malaysia’s coastal water bodies, *M.kertehensis* sp. nov. is similar to *M.moribidii* (type locality: Morib, Malaysia) in lacking eyes. Table 5 lists the characteristics of Group A species, such as the presence or absence of peduncle in the prostomial appendages, the number of types of pectinate chaetae, chaetiger from where the branchiae commence and finish, number of branchial filaments and subacicular hooks and all differ from the new species. *Marphysakertehensis* sp. nov. has three types of pectinate chaetae (types 1, 2, 4) but lacks any wide anodont chaetae (types 6, 7, 8), while *M.moribidii* has four types, including wide anodont (types 1, 4, 5, 8). Although they all have the same type of pectinate branchiae and the chaetiger where the branchiae emerge, *M.moribidii* (TL: 333 mm) has a wider range variation of chaetiger where the branchiae emerge; they occur from chaetiger 35 (4–63) whereas in *M.kertehensis* sp. nov. (TL: 413 (173–295) mm), the branchiae are present from chaetiger 41 (27–58). There are no subacicular hooks present in all specimens of *M.kertehensis* sp. nov., but there are a few subacicular hooks present in the paratype of *M.moribidii*AM W.38690. Additionally, *M.kertehensis* sp. nov. has a black dot at the base of dorsal cirri in median and posterior chaetigers, possibly a reservoir of blood to irrigate the branchiae, which is absent in *M.moribidii*. It is worth mentioning that comparisons between the two species were based only on morphological features as there is no sequence data published for *M.moribidii*. Furthermore, each species lives in a different habitat. *Marphysakertehensis* sp. nov. was found in the driftwood within the mangrove area dominated by *Exoecariaagallocha*, meanwhile *M.moribidii* inhabits mangrove forest with *Rhizophora* spp., *Avicenniaalba* and *Sonneratiacaseolaris* (Idris et al. 2014).

**Table 5.** Morphological features comparison between *Marphysa* Group A (Mossambica) described in this study and species occurring within Malaysian water bodies. The features for new species are based on holotype, with variation in parentheses for paratypes. Abbreviations: MF: maxillary formula, roman numerals refer to number of maxilla; PR-I: first peristomial ring; PR-II: second peristomial ring; p/a: present/absent; NIA: no information available. The major feature’s differences between the species are mark with asterisk (*).

Morphological feature	*M.moribidii* Idris, Hutchings & Arshad, 2014	*M.kertehensis* sp. nov.
Source of Information	Paratypes AM W.38690; additional material (Idris et al. 2014)	Holotype UMTAnn 2181 (this study)
Size (mm): L10, W10	12.2–20, 6.3–8.2	12 (6–10.8) , 4.8 (3.15–5.1)
Prostomium: shape	Bilobed	Bilobed
Palps: reaching	PR-II	PR-I
Lateral antennae:reaching	PR-II or Chaetiger 1	PR-II
Median antennae: reaching	Chaetiger 1 or 2	PR-II
Peduncle in prostomial appendages*	Present	Absent
Eyes	Absent	Absent
MF: MII, MIII, MIV*	5–6+4–6, 7-8+0, 6+8–10	5+5 (4–5), 8 (7–8)+0, 3 (3–4)+9 (8–9)
Branchiae: shaped	Pectinate	Pectinate
Branchiae: start chaetiger; last chaetiger before pygidium*	27–39; 15–37	41 (27–58), until pygidium
Branchial filaments: numbers	7–10	9
Dorsal cirri: shaped	Conical	Digitiform
Prechaetal lobe: shaped	Transverse fold	Transverse fold
Chaetal lobe: shaped	Rounded	Conical and directed to ventral cirri, conical
Aciculae: shape; colour	Blunt, dark	Black, dark and translucent at distal end
Subacicular limbate chaetae: (p/a); distribution	Present; all chaetigers	Present; all chaetigers
Pectinate chaetae: number of type*	4	3
Subacicular hook: shape; colour*	Bidentate, translucent	No subacicular hook
Subacicular hook: start chaetiger*	56–65	No subacicular hook
Subacicular hook: distribution*	Scattered	No subacicular hook

#### ﻿ Marphysa merchangensis sp. nov.

Taxon classificationAnimaliaEunicidaEunicidae

37BB3E05-77F6-50AD-9CA2-0FA6E8FDE9F4

https://zoobank.org/AD77E9BF-8D3D-458F-8AEA-BAC2E7AAFCB7

**Figure 8.**
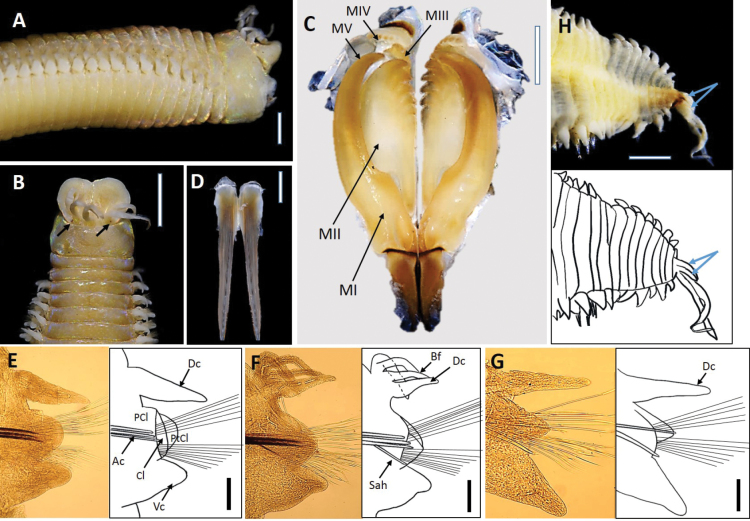
*Marphysamerchangensis* sp. nov. Holotype UMTAnn 2149 (**B–H**), paratype UMTAnn 2148 (**A**). Light microscopy images and digital drawing **A** anterior end, lateral view **B** anterior end, dorsal view. Arrows indicate eyes **C** maxillae, dorsal view **D** mandibles, dorsal view **E** parapodium, chaetiger 10 **F** parapodium, chaetiger 134 **G** parapodium, chaetiger 250 **H** posterior segments and pygidium, ventral view. Arrows show the short pair of pygidial cirri. Abbreviations; MI–MV: maxillae I–V, Ac: aciculae, Dc: dorsal cirrus, Vc: ventral cirrus, PCl: prechaetal lobe, Cl: chaetal lobe, PtCl: postchaetal lobe, Sah: subacicular hook, Bf: branchial filament. Scale bars: 1 mm (**A–D, H**); 0.1 mm (**E–G**).

**Figure 9.**
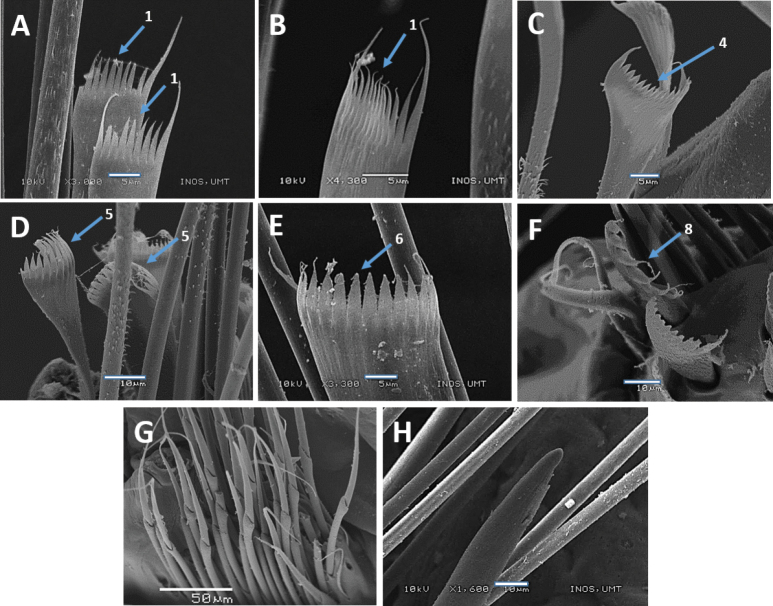
SEM images of *Marphysamerchangensis* sp. nov. Holotype UMTAnn 2149 (**A, B, E, H**), paratype AM W.54044 (**C, D, F, G**) **A, B** pectinate chaetae, chaetiger 10 **C** pectinate chaetae, chaetiger 164 **D** pectinate chaetae, chaetiger 245 **E** pectinate chaetae, chaetiger 250 **F** pectinate chaetae, chaetiger 265 **G** spiniger chaetae, chaetiger 10 **H** subacicular hook, chaetiger 128. Numbers denoted by arrows indicate the type of pectinate chaetae; 1. Thin, narrow isodont; 4, 5. Thick, wide isodont; 6. Thick, narrow anodont; 8. Thick, wide anodont. Scale bars: 5 µm (**A–C, E**); 10 µm (**D, F, H**); 50 µm (**G**).

**Figure 10.**
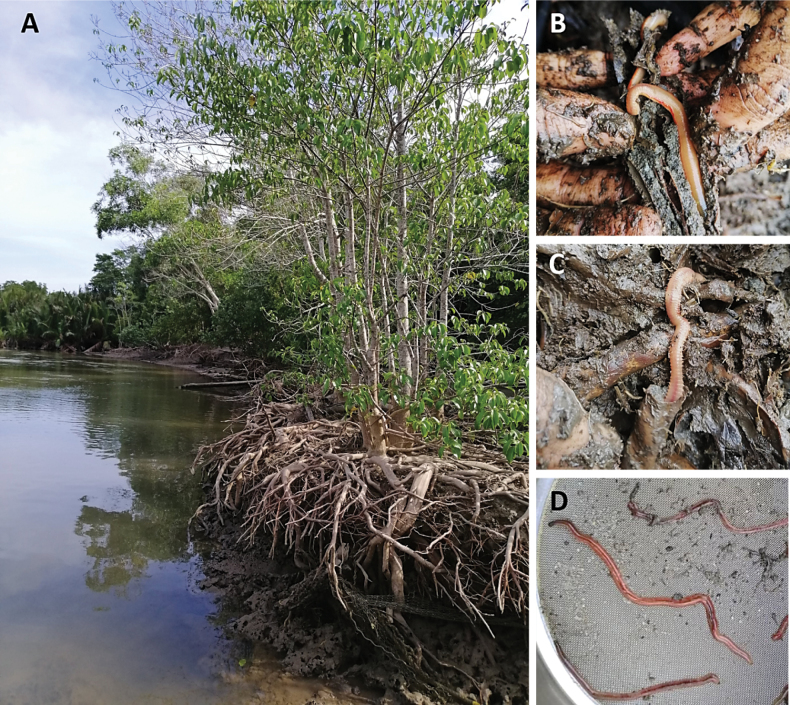
Sampling site in Merchang mangrove estuary **A** habitat of *M.merchangensis* sp. nov. **B–C** worm found in decayed root of *Exoecariaagallocha* (Malay: Bebuta) **D** live worms.

Figs 1 , 2 , 8 , 9 , 10

##### Material examined.

***Holotype*.** UMTAnn 2149, complete, antero-ventrally dissected, some parapodia mounted for SEM. ***Paratypes*.**AM W.54044, complete, some parapodia mounted for SEM. LACM-AHF 13494 to 13496, complete, some parapodia removed; ZRC.ANN.1604 to 1606, complete, some parapodia removed; SAM-MB-A096021, complete, some parapodia removed. All material was collected from the east coast of Peninsular Malaysia, Terengganu, Merchang mangrove estuary (05°01.393'N, 103°17.994'E), October 2021.

##### Diagnosis.

Prostomium completely bilobed, five prostomial appendages without articulations; eyes present. Peristomium without peristomial cirri. Maxillary apparatus with four pairs of maxillae, an unpaired on the left side, MI with falcal arch extended at sub-right angle, basal outer edge arched, basal inner edge lacking curvature. MII with triangular teeth and without attachment lamella. MIII slightly curved, with equal-sized triangular teeth, without attachment lamella. MIV with rectangular and curved attachment lamella. Branchiae distributed along entire body. Dorsal cirri without articulations; postchaetal lobe well developed in anterior regions. Ventral cirri with swollen, inflated base. Sub-aciculae black, blunt, and translucent at distal end, pale brown in posterior-most parapodia. Supra-acicular chaetae include limbate, pectinate thin, narrow isodont with short and slender inner teeth, pectinate thick, wide isodont with short or long and slender inner teeth, and pectinate thick, narrow and wide anodont with long and thick inner teeth. Subacicular chaetae include only compound spinigers. Subacicular hook unidentate throughout chaetigers. Pygidium with two pairs of anal cirri, without articulation.

##### Description

**(based on holotype, with variation in parentheses for paratypes).** Preserved specimen beige (Fig. 8A), 257 (165–294) chaetigers, 94 mm (37–144 mm) long, L10 - 5.25 mm (3.45–5.85 mm), W10 - 2.85 mm (1.95–3.15 mm), excluding parapodia. Anterior region of body with dorsum convex and flat ventrum, without groove (Fig. 8A); body depressed from chaetiger 25, elongated and tapering at distal end. Live specimens pink with red branchiae (Fig. 10D).

Prostomium bilobed, anteriorly rounded with two dorsoventrally flattened lobes with an anterior notch between them (Fig. 8A, B). Prostomial appendages in a semicircle, median antenna isolated by a gap (Fig. 8B). Palps reach middle of second peristomial ring; lateral antennae reaching chaetiger 2; median antenna reaching chaetiger 3. Palpophores and ceratophores ring-shaped, short, and thick; palpostyles and ceratostyles tapering, and slender. Prostomial appendage peduncles absent. A pair of faded brown eyes present at posterior base of prostomium, between palps and lateral antennae (Fig. 8B). Peristomium larger and wider than prostomium; first ring is 2.5× longer than second ring, separation between rings distinct on all sides.

Maxillae pale brown (Fig. 8C), and maxillary formula as follows: MF = 1+1, 5 (4–5)+5 (5–6), 7 (6–7)+0, 4 (4–5)+8 (5–8), 1+1. Maxillary carrier ~ 2.5× shorter than MI, rectangular anteriorly, triangular posteriorly. MI forceps-like, without attachment lamellae, falcal arch extended at sub-right angle, basal outer edge arched, basal inner edge lacking a curvature. Closing system ~ 3× shorter than MI. Ligament between MI and MII pale brown. MII without attachment lamella, teeth triangular, distributed on < 1/2 length of the plate. Ligament between MII and MIII pale brown. MIII single, longer than left MIV slightly curved, with equal-sized triangular teeth, without attachment lamella. Left MIV short (< 1/2 the size of right MIV) with rectangular attachment lamellae. Right MIV long with curved attachment lamellae, teeth triangular, decreasing in size and teeth curved posteriorly. MV paired. Mandible pale brown, with concentric stripes, longer than MI; cutting plates whitish (Fig. 8D).

First few parapodia located ventrolaterally but gradually becoming dorsolateral in subsequent segments. Chaetal lobes rounded in anterior and posterior chaetigers, conical in median chaetigers (Fig. 8E–G). Prechaetal lobe shorter than chaetal lobe throughout body. Postchaetal lobe digitiform in first three chaetigers then rounded thereafter; longer than chaetal lobe in median chaetigers onwards, become shorter and absent in the posterior-most chaetigers. Dorsal cirri digitiform and slender, longer than ventral cirri anteriorly, slightly longer or similar from mid-body towards posterior-most chaetigers (Fig. 8E–G). Ventral cirri thumb-shaped with rounded wide tips in first few chaetigers, basally inflated with digitiform tip from chaetiger 15, and gradually becoming conical posteriorly (Fig. 8E–G). Branchiae pectinate, from chaetiger 24 (16–27) and continuing to last ~ 10 chaetigers, branchial filaments 4× longer than dorsal cirri where best developed; number of filaments increasing from three anteriorly to six in mid-body, decreasing to one in last several chaetigers.

Notoaciculae absent, neuroaciculae black, blunt, and translucent at distal end along most of body, pale brown in posterior-most parapodia; ~ 2 or 3 per parapodium in anterior, one per parapodium in median and posterior chaetigers (Fig. 8E–G). Supra-acicular chaetae with limbate capillaries and pectinates. Five types of pectinate chaetae present (types 1, 4, 5, 6, 8) (see Fig. 2): type 1: thin, narrow isodont with 7–12 short and slender inner teeth, outer teeth longer, but with varying lengths, present in anterior and median body region (Fig. 9A, B); type 4: thick, wide isodont with 12–15 short and slender inner teeth, present only in median and posterior region (Fig. 9C); type 5: thick, wide isodont, with 15–18 long and slender inner teeth, only present in posterior region (Fig. 9D); type 6: thick, narrow anodont with 11 or 12 long thick teeth, only present in posterior region (Fig. 9E); type 8: thick, wide anodont, with five inner long and thick teeth, only present in the posterior region (Fig. 9F). Subacicular chaetae with compound spinigers (Fig. 9G). Subacicular hooks pale brown, translucent at distal end, emerge from chaetiger 37 (26–42) and then present on all chaetigers, one per parapodium; subacicular hooks unidentate throughout chaetigers (Fig. 9H). Pygidium with crenulated margin, with two pairs of tapering pygidial cirri attached to ventral side of pygidium, dorsal pair ~ 4× longer than ventral (Fig. 8H).

##### Etymology.

The name denotes the type locality (Merchang estuary) where the specimens were collected.

##### Type locality.

South China Sea, Malaysia, east coast of Peninsular, Terengganu, Merchang mangrove estuary (see Fig. 1).

##### Distribution.

Known only from the type locality and Setiu Wetlands, Terengganu, Malaysia.

##### Habitat.

Gravelly and slightly gravelly sand (Table 4), burrowing in decayed roots of the mangrove *E.agallocha* (Malay: Bebuta) (Fig. 10A–C), burrowing in the sediments within an area populated with *Taliparititiliaceum* (Fig. 13C) with salinity 26‰ during spring low tide.

##### Remarks.

With the presence of only compound spinigers along the whole body and branchiae along most of the body, *Marphysamerchangensis* sp. nov. belongs to the *Marphysa* Group B (Sanguinea). Other *Marphysa* species from Sanguinea-group occurring in the same water body (South China Sea) as *M.merchangensis* sp. nov. are *M.setiuense* sp. nov., *M.hongkongensa* Wang, Zhang & Qiu, 2018 (type locality: Hong Kong), *M.iloiloensis* Glasby, Mandario, Burghardt, Kupriyanova, Gunton & Hutchings, 2019 (type locality: Philippines), *M.multipectinata* Liu, Hutchings & Sun, 2017 (type locality: Shimen, Taiwan of China), *M.orientalis* Treadwell, 1936 (type locality: Xiamen, China), *M.tribranchiata* Liu, Hutchings & Sun, 2017 (type locality: Wanli, Taiwan of China), and *M.tripectinata* Liu, Hutchings & Sun, 2017 (type locality: Beihai, China).

*Marphysamerchangensis* sp. nov. is similar to *M.setiuense* sp. nov. in having a pair of eyes and the absence of peduncle on the prostomial appendages. However, they can be differentiated by the number of types of pectinate chaetae, maxillary formula, chaetiger on which the branchiae and subacicular hooks occur, shape of dorsal cirri, chaetal lobes and subacicular hooks. Number of types of pectinate chaetae in *M.merchangensis* sp. nov. is five (types 1, 4, 5, 6, 8), whereas in *M.setiuense* sp. nov. there are four (types 1, 2, 7, 8), and they lack the thick, wide isodont pectinate chaetae (types 4, 5). *Marphysamerchangensis* sp. nov. (L10: 5.25 (3.45–5.85) mm) has more denticles on MIII 7 (6–7)+0 compared to *M.setiuense* sp. nov. (L10: 2.7 (2.85–4.8) mm) which has MIII: 5 (4–6)+0. Branchiae and subacicular hook of *M.merchangensis* sp. nov. occur later (chaetiger 24 (16–27) and 37 (26–42)), respectively) compared to *M.setiuense* sp. nov., where they occur from chaetiger 20 (15–25) and 25 (21–38), respectively. *Marphysamerchangensis* sp. nov. has digitiform dorsal cirri along the whole body, while *M.setiuense* sp. nov. has both thumb-shaped and digitiform dorsal cirri. *Marphysamerchangensis* sp. nov. has rounded shaped chaetal lobe in the anterior and posterior, and conical in the median region, whereas *M.setiuense* sp. nov. has rounded chaetal lobes on all parapodia. Finally, *M.merchangensis* sp. nov. has unidentate subacicular hook, whereas *M.setiuense* sp. nov. has unidentate and a few bidentate subacicular hooks present in posterior chaetigers.

*Marphysamerchangensis* sp. nov. and *M.hongkongensa* can be differentiated by the presence or absence of eyes, number of types of pectinate chaetae, maximum number of branchial filaments, and the shape of subacicular hooks. *Marphysamerchangensis* sp. nov. has a pair of eyes but they are absent in *M.hongkongensa*. *Marphysamerchangensis* sp. nov. has five types of pectinate chaetae (types 1, 4, 5, 6, 8) compared to four types present in *M.hongkongensa* (types 1, 2, 7, 8). *Marphysahongkongensa* lacks thick, wide isodont and thick, narrow anodont pectinate chaetae (types 4, 5, 6) which are present in the new species. The maximum number of branchial filaments in *M.merchangensis* sp. nov. (L10: 5.25 (3.45–5.85) mm) is six and they begin from chaetiger 24 (16–27) whereas *M.hongkongensa* (L10: 3.3–7 mm) has a maximum of ten branchial filaments, beginning from chaetiger 15–35. Finally, *M.merchangensis* sp. nov. only has unidentate subacicular hooks while both unidentate and bidentate subacicular hooks are present in *M.hongkongensa*.

*Marphysamerchangensis* sp. nov. is similar to *M.iloiloensis* and *M.multipectinata* in having a pair of eyes. However, they can be distinguished by the number of types of pectinate chaetae present, the chaetiger on which branchiae and subacicular hooks occur, number of branchial filaments, shape of subacicular hooks and the maxillae formula. *Marphysamerchangensis* sp. nov. has five types of pectinate chaetae (types 1, 4, 5, 6, 8) whereas *M.iloiloensis* and *M.multipectinata* have three (types 1, 4, 6) and four (types 1, 4, 7, 8) respectively. *Marphysamerchangensis* sp. nov. and *M.iloiloensis* have the same type of pectinate branchiae (beginning on the same chaetiger with different range of variation) (chaetiger 24 (16–27) for the new species, and chaetiger 19 (16–20) for *M.iloiloensis*). The maximum number of branchial filaments in *M.merchangensis* sp. nov. (TL: 94 (37–144) mm) is six, while *M.iloiloensis* (TL: 99 (95–165+) mm) has a maximum of seven branchial filaments. *Marphysamultipectinata* (L10: 13.9 mm) has palmate branchiae with maximum of five branchial filaments from chaetiger 32. Finally, all these species have different formulae for MII, MIII and MIV (see Table 6).

**Table 6.** Morphological features comparison between *Marphysa* Group B (Sanguinea) described in this study and species occurring within Malaysian water bodies (South China Sea). The features for new species are based on the holotype, with variation in parentheses for paratypes. Abbreviations: MF: maxillary formula, roman numerals refer to number of maxilla; PR-I: first peristomial ring; PR-II: second peristomial ring; p/a: present/absent; NIA: no information available. The major differences between the species are marked with asterisk (*).

Morphological feature	*M.hongkongensa* Wang, Zhang & Qiu, 2018	*M.iloiloensis* Glasby, Mandario, Burghardt, Kupriyanova, Gunton & Hutchings, 2019	*M.multipectinata* Liu, Hutchings & Sun, 2017	*M.orientalis*, Treadwell, 1936	*M.tribranchiata* Liu, Hutchings & Sun, 2017	*M.tripectinata* Liu, Hutchings & Sun, 2017	*M.merchangensis* sp. nov.	*M.setiuense* sp. nov.
Source of Information	Holotype: SWIMS-ANN-18-012; Paratypes: SWIMS-ANN-18-013- (Wang et al. 2018)	Holotype: NTM W29624; Paratypes: NTM W29619 – NTM29623 (Glasby et al. 2019)	Holotype: ASIZW0000345-1 (Liu et al. 2017)	Type: USNM. No. 20114 (Treadwell 1936)	Holotype: ASIZW0000348-2 (Liu et al. 2017)	Holotype: AM W.49069 (Liu et al. 2017)	Holotype: UMTAnn 2149 (this study)	Holotype: UMTAnn 2177 (this study)
Size (mm): L10, W10	3.3–7.0, 2.2–5.3	NIA, 2.6	13.9, 5.7	NIA	8.7, 3.9	12.7, 5.95	5.25 (3.45–5.85), 2.85 (1.95–3.15)	2.7 (2.85–4.8), 1.8 (1.65–2.55)
Prostomium: shape	Bilobed	Bilobed	Bilobed	Bilobed	Bilobed	Bilobed	Bilobed	Bilobed
Palps: reaching	Chaetiger 1	Chaetiger 1	PR-I	NIA	Chaetiger 1	PR-I	PR-II	Chaetiger 3
Lateral antennae:reaching	Chaetiger 1	Chaetiger 1 or 2	PR-I	NIA	Chaetiger 2	PR-II	Chaetiger 2	Chaetiger 4
Median antennae: reaching	Chaetiger 1 or 2	Chaetiger 1 or 2	PR-II	NIA	Chaetiger 3	Chaetiger 1	Chaetiger 3	Chaetiger 5
Peduncle in prostomial appendages	Absent	Absent	Absent	Absent	Absent	Absent	Absent	Absent
Eyes*	Absent	Present	Present	Absent	Absent	Absent	Present	Present
MF: MII, MIII, MIV*	5-6+5–6, 7+0, 4+8	4+5, 4–5+0, 3–4+5–6	3+3, 4+0, 4+5	3+3, 4+0, 4+3	4+4, 5+0, 4+8	5+5, 5+0, 4+8	5 (4–5)+5 (5–6), 7 (6–7)+0, 4 (4–5)+8 (5–8)	5 (4–5)+5 (4–6), 5 (4–6)+0, 3 (3–4)+6 (7–8)
Branchiae: shape	Pectinate	Pectinate	Palmate	NIA	Pectinate	Pectinate	Pectinate	Pectinate
Branchiae: start chaetiger; last chaetiger before pygidium	15–35; until pygidium	16–20; until pygidium	32; end at chaetiger 281	45; end ~30 last chaetiger	26; end at chaetiger 181	15; end at chaetiger 399	24 (16–27); end ~10 last chaetiger	20 (15–25); until pygidium
Branchial filaments: numbers	5–10	6–7	5	3	3	8	6	5
Dorsal cirri: shaped	Conical	Conical	NIA	Conical	NIA	NIA	Digitiform	Thumb-shape, digitiform
Prechaetal lobe: shaped	Transverse fold	Transverse fold	NIA	NIA	NIA	NIA	Transverse fold	Transverse fold
Chaetal lobe: shaped	Rounded	Rounded	NIA	Rounded	NIA	NIA	Rounded and conical	Rounded
Aciculae: shape; colour	NIA; black with paler tips	Blunt; black with paler tips	NIA; brown	Blunt; black	NIA; brown	NIA; black	Blunt; black and translucent at distal end	Blunt; black and translucent at distal end
Subacicular limbate chaetae: (p/a); distribution	Absent; all chaetigers	Absent; all chaetigers	Absent; all chaetigers	Absent; all chaetigers	Absent; all chaetigers	Absent; all chaetigers	Absent; all chaetigers	Absent; all chaetigers
Pectinate chaetae: number of type*	4	3	4	NIA	3	3	5	4
Subacicular hook: shape; colour*	Unidentate and bidentate; amber	Unidentate; amber to black	Unidentate and bidentate; yellow	Unidentate; NIA	Unidentate and bidentate; brown	Unidentate	Unidentate; light brown and translucent at distal end	Unidentate and bidentate; light brown and translucent at distal end
Subacicular hook: start chaetiger*	26–58	30–38	20	Present in posterior region (No information on start chaetiger)	20	170	37 (26–42)	25 (21–38)
Subacicular hook: distribution	Continuous	Continuous	Continuous	NIA	Continuous	Continuous	Continuous	Continuous

The other species from the Sanguinea complex, *M.tribranchiata* and *M.tripectinata* differ from *M.merchangensis* sp. nov. by the absence of eyes. Both *M.tribranchiata* and *M.tripectinata* have three types of pectinate chaetae, whereas *M.merchangensis* sp. nov. has five types. *Marphysatribranchiata* lacks thick, wide isodont and thick, narrow anodont pectinate chaetae (types 4, 6), while *M.tripectinata* lacks thin, narrow isodont pectinate chaetae (type 1) which are present in the new species (types 1, 4, 5, 6, 8). While *M.merchangensis* sp. nov. and *M.tripectinata* only have unidentate subacicular hooks, they begin much later (chaetiger 170) in the latter species. *Marphysatribranchiata* has both unidentate and bidentate subacicular hooks whereas only unidentate hooks are present in *M.merchangensis* sp. nov. The maximum number of branchiae filaments present in *M.tribranchiata* (L10: 8.7 mm) and *M tripectinata* (L10: 12.7 mm) are three and eight respectively, differs from *M.merchangensis* sp. nov. (L10: 5.25 (3.45–5.85) mm), which has a maximum of six.

Finally, *M.merchangensis* sp. nov. is similar to *M.orientalis* by having unidentate subacicular hooks. *Marphysamerchangensis* sp. nov. has a pair of eyes and two pairs of anal cirri, while *M.orientalis* has no eyes and only one pair of anal cirri. Also, branchiae in *M.merchangensis* sp. nov. begin earlier from chaetiger 24 (16–27) compared to *M.orientalis* (chaetiger 45). The maximum number of branchial filaments in *M.merchangensis* sp. nov. is six, while *M.orientalis* has a maximum of three branchial filaments. Nevertheless, the original description of *M.orientalis* is incomplete and does not include certain important features such as the number and type of pectinate chaetae. Fresh material of *M.orientalis* should be collected and redescribed from the type locality at Gulf of Mannar, Sri Lanka.

#### ﻿ Marphysa setiuense sp. nov.

Taxon classificationAnimaliaEunicidaEunicidae

01A9E8EE-C19B-5AB1-94C5-0570A564565C

https://zoobank.org/46E5DB0B-5F17-45D6-A57E-56AD91799411

**Figure 11.**
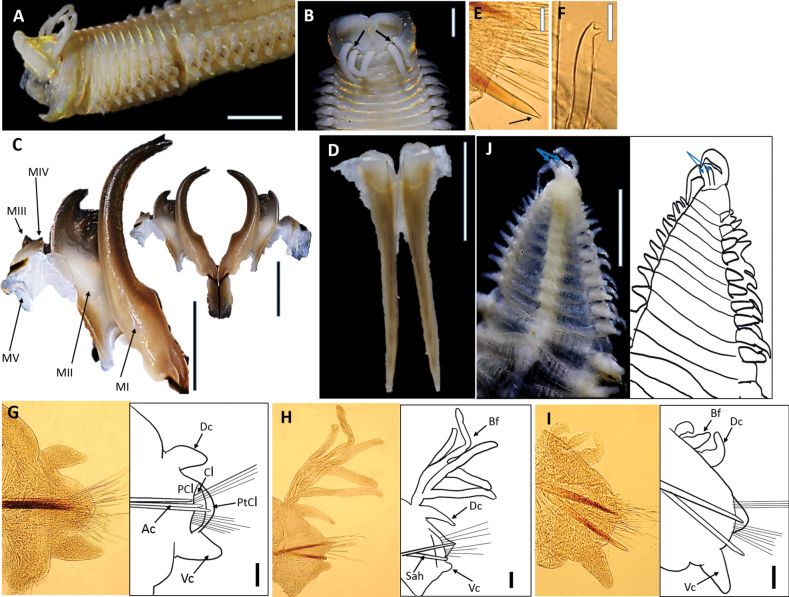
*Marphysasetiuense* sp. nov. Holotype UMTAnn 2177 (**A–J**). Light microscopy images and digital drawing **A** anterior end, lateral view **B** anterior end, dorsal view. Arrows indicate eyes **C** maxillae, dorsal view **D** mandibles, dorsal view **E** unidentate hook, chaetiger 136 **F** bidentate hooded hook, chaetiger 140 **G** parapodium, chaetiger 10 **H** parapodium, chaetiger 77 **I** parapodium, chaetiger 136 **J** posterior segments and pygidium, dorsal view. Arrows show the short pair of pygidial cirri. Abbreviations; MI–MV: maxillae I–V, Ac: aciculae, Dc: dorsal cirrus, Vc: ventral cirrus, PCl: prechaetal lobe, Cl: chaetal lobe, PtCl: postchaetal lobe, Sah: subacicular hook, Bf: branchial filament. Scale bars: 1 mm (**A, C, D, J**); 1 mm (**B**); 20 µm (**E–F**); 0.1 mm (**G–I**).

**Figure 12.**
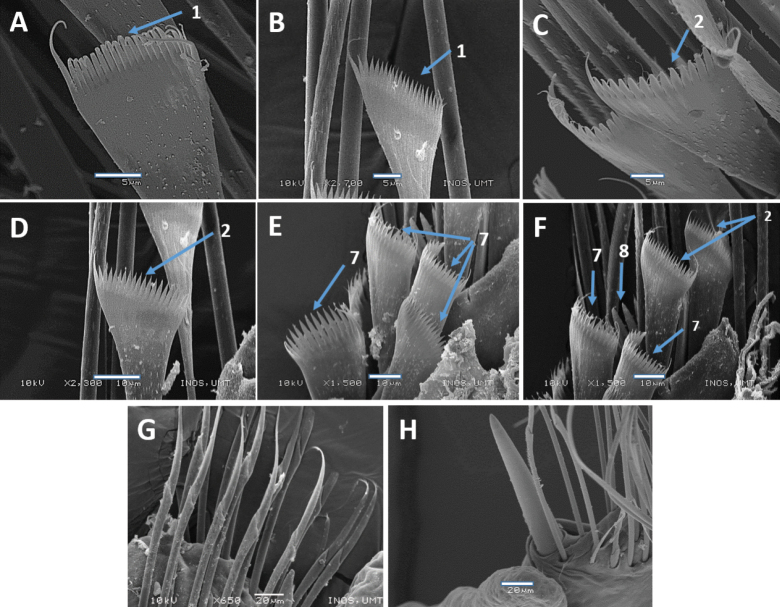
SEM images of *Marphysasetiuense* sp. nov. Holotype UMTAnn 2177 (**B, D–G**), paratype AM W.54050 (**A, C, H**) **A** pectinate chaetae, chaetiger 30 **B** pectinate chaetae, chaetiger 77 **C** pectinate chaetae, chaetiger 203 **D** pectinate chaetae, chaetiger 77 **E, F** pectinate chaetae, chaetiger 136 **G** spiniger chaetae, chaetiger 25 **H** subacicular hook, chaetiger 183. Numbers denoted by arrows indicate the type of pectinate chaetae; 1. Thin, narrow isodont; 2. Thin, wide isodont; 7, 8. Thick, wide anodont. Scale bars: 5 µm (**A–C**); 10 µm (**D–F**); 20 µm (**G, H**).

**Figure 13.**
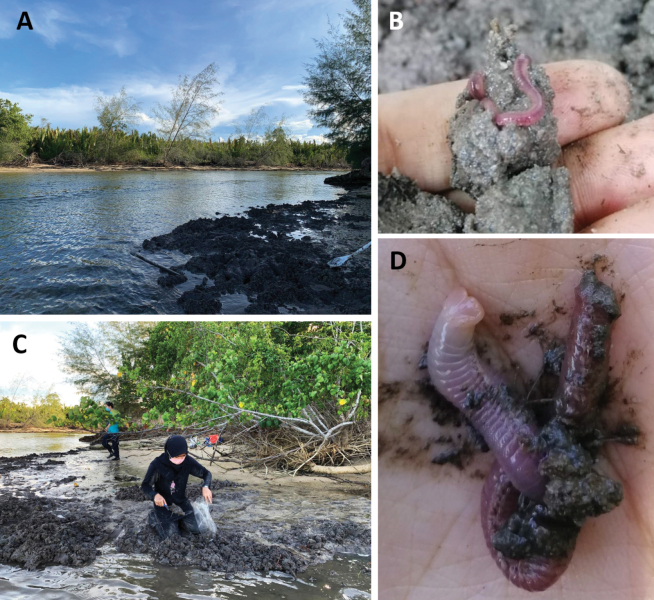
Sampling in Setiu Wetlands (river area) **A, C** habitat of *M.setiuense* sp. nov. and *M.merchangensis* sp. nov. within *Taliparititiliaceum***B***Marphysasetiuense* sp. nov. in situ **D** live *M.setiuense* sp. nov.

Figs 1 , 2 , 11 , 12 , 13

##### Material examined.

***Holotype*.** UMTAnn 2177, complete, antero-ventrally dissected, some parapodia mounted for SEM. ***Paratypes*.**AM W.54050, complete, some parapodia mounted for SEM. LACM-AHF 13497 to 13499, complete, some parapodia removed; ZRC.ANN.1607 to 1609, complete, some parapodia removed. All material was collected from the east coast of Peninsular Malaysia, Terengganu, Setiu Wetlands (05°39.183'N, 102°45.194'E), October 2021.

##### Diagnosis.

Prostomium completely bilobed, five prostomial appendages without articulations; eyes present. Peristomium without Peristomial cirri. Maxillary apparatus with four pairs of maxillae, an unpaired on the left side, MI with falcal arch extended at sub-right angle, basal outer edge arched, basal inner edge lacking curvature. MII with triangular teeth and without attachment lamella. MIII slightly curved, with equal-sized triangular teeth, without attachment lamella, MIV with curved attachment lamella. Branchiae distributed along entire body. Dorsal cirri without articulations; postchaetal lobe well developed in anterior regions. Ventral cirri with swollen, inflated base. Sub-aciculae black, blunt, and translucent at distal end, pale brown in posterior-most parapodia. Supra-acicular chaetae include limbate, pectinate thin, narrow and wide isodont with short and slender inner teeth, and pectinate thick, wide anodont with long and slender or thick inner teeth. Subacicular chaetae include only compound spinigers. Subacicular hook unidentate, and a few bidentate present in posterior chaetigers. Pygidium with two pairs of anal cirri, without articulation.

##### Description

**(based on holotype, with variation in parentheses for paratypes).** Preserved specimens beige (Fig. 11A), ~ 154 (141–259) chaetigers, ~ 51 mm (27–75 mm) long, L10 - 2.7 mm (2.85–4.8 mm), W10 - 1.8 mm (1.65–2.55 mm), excluding parapodia. Anterior region of the body with dorsum convex and flat ventrum, without groove; body depressed from chaetiger 11, elongated and tapering at the distal end. Live specimens pink (Fig. 13B, D).

Prostomium bilobed, anteriorly rounded with two dorsoventrally flattened lobes separated by an anterior notch (Fig. 11A, B). Prostomial appendages in a semicircle, median antenna isolated by a gap (Fig. 11B). Palps reaching chaetiger 3; lateral antennae reaching chaetiger 4; median antenna reaching chaetiger 5. Palpophores and ceratophores ring-shaped, short, and thick; palpostyles and ceratostyles tapering and slender. Prostomial appendage peduncles absent. Pair of faded brown eyes at posterior base of prostomium, between palps and lateral antennae. Peristomium similar in size (width and length) to prostomium; the first ring is 1.5× longer than second ring, and separation between rings distinct on all sides.

Maxillae dark brown (Fig. 11C) and maxillary formula as follows: MF = 1+1, 5 (4–5)+5 (4–6), 5 (4–6)+0, 3 (3–4)+6 (7–8), 1+1. Maxillary carrier ~ 2.4× shorter than MI, rectangular anteriorly, triangular posteriorly. MI forceps-like, without attachment lamellae, falcal arch extended at sub-right angle, basal outer edge arched, basal inner edge lacking a curvature. Closing system ~ 5× shorter than MI. Ligament between MI and MII pale brown. MII without attachment lamella, teeth triangular, distributed along half of plate length. Ligament between MII and MIII pale brown. MIII single, longer than left MIV, slightly curved, with equal-sized triangular teeth, without attachment lamella. Left MIV short (< 1/2 the size of right MIV) with curved attachment lamellae. Right MIV long, with teeth triangular with curved attachment lamellae, decreasing in size and teeth curved posteriorly. MV paired. Mandibles dark brown (Fig. 11D), with concentric stripes; longer than MI; cutting plates whitish.

First parapodia occur ventrolaterally, gradually becoming dorsolateral in following segments. Chaetal lobes rounded in all chaetigers (Fig. 11G–I). Prechaetal lobe shorter than chaetal lobe along whole body. Postchaetal lobe digitiform in first three chaetigers and rounded thereafter; conical and longer than chaetal lobe in median and posterior chaetigers, becoming shorter and absent in the posterior-most chaetigers. Dorsal cirri thumb-shaped with digitiform tips, shorter than ventral cirri in anterior, digitiform with slender and tapering tips; slightly longer or similar length from mid-body onwards and shorter in posterior-most chaetigers (Fig. 11G–I). Ventral cirri thumb-shaped with digitiform tips in the first few chaetigers, basally inflated with digitiform tip from chaetiger 15 onwards, and gradually becoming conical posteriorly (Fig. 11G–I). Branchiae pectinate, from chaetiger 20 (15–25) and continuing to near the end (~ 8 last chaetigers without branchiae), branchial filament 4× longer than dorsal cirri where best developed; number of filaments increasing from two anteriorly to five in mid-body, decreasing to one in last several chaetigers.

Notoaciculae absent, neuroaciculae black, blunt, and translucent at distal end on most of body, pale brown in posterior-most parapodia; ~ 2 or 3 per parapodium in anterior, one per parapodium in median and posterior chaetigers (Fig. 11G–I). Supra-acicular chaetae with limbate capillaries and pectinates. Four types of pectinate chaetae were identified (types 1, 2, 7, 8) (see Fig. 2): type 1: thin, narrow isodont with ~ 18–22 short and slender inner teeth, outer teeth longer, but of varying lengths, present in anterior and median body region (Fig. 12A, B); type 2: thin, wide isodont with 14–21 short and slender teeth, outer teeth same length as inner teeth, present only in anterior and posterior region (Fig. 12C, D); type 7: thick, wide anodont with 15–18 long and slender inner teeth, only present in posterior region (Fig. 12E, F); type 8: thick, wide anodont, with seven inner long and thick teeth, only present in posterior region (Fig. 12F). Subacicular chaetae with compound spinigers (Fig. 12G). Subacicular hooks unidentate (Figs 11E, 12H), pale brown, translucent at distal end, commencing from chaetiger 25 (21–38) and then present on all subsequent chaetigers, one per parapodium and with a few bidentate hooks in posterior chaetigers (Fig. 11F). Pygidium with crenulated margin, with two pairs of tapering pygidial cirri attached to ventral side of pygidium, dorsal pair ~ 4× longer than ventral one (Fig. 11J).

##### Etymology.

The name refers to the type locality Setiu Wetlands.

##### Type locality.

South China Sea, Malaysia, east coast of Peninsular, Terengganu, Setiu Wetlands (see Fig. 1).

##### Distribution.

Known only from the type locality.

##### Habitat.

Slightly gravelly sand sediment (Table 4), burrowing in decayed roots of mangrove trees (*Sonneratia* spp.) and area within *Taliparititiliaceum* (Fig. 13A–C), with salinity 26‰ during spring low tide.

##### Remarks.

With the presence of only compound spiniger along the whole body and branchiae along most of the body, *Marphysasetiuense* sp. nov. belongs to Group B (Sanguinea). As mentioned earlier, there are seven other Sanguinea-group *Marphysa* species described from the South China Sea; *M.merchangensis* sp. nov., *M.hongkongensa*, *M.iloiloensis*, *M.multipectinata*, *M.orientalis*, *M.tribranchiata* and *M.tripectinata*. The most morphologically-similar species to *M.setiuense* sp. nov. is *M.hongkongensa*. Both species have four types of pectinate chaetae (two isodont and two anodont; types 1, 2, 7, 8) and have both unidentate and bidentate subacicular hooks in posterior chaetigers. However, they differ in the number of branchial filaments and the distribution of branchiae. *Marphysasetiuense* sp. nov. (L10: 2.7 (2.85–4.8) mm) has a maximum of five branchial filaments while *M.hongkongensa* (L10: 3.3–7 mm) has up to ten. Also, the species have different maxillae formulae. *Marphysasetiuense* sp. nov. has fewer denticles on MIII (5 (4–6)+0) compared to *M.hongkongensa* which has MIII (7+0) (see Table 6).

*Marphysasetiuense* sp. nov. is similar to *M.iloiloensis* and *M.multipectinata* in having a pair of eyes, but they can be distinguished by the number of types of pectinate chaetae, the chaetiger on which branchiae and subacicular hooks begin, number of branchial filaments, shape of subacicular hooks and maxillae formula. *Marphysasetiuense* sp. nov. has four types of pectinate chaetae (types 1, 2, 7, 8) compared to three types present in *M.iloiloensis* (types 1, 4, 6). *Marphysamultipectinata* also has four types of pectinate chaetae (types 1, 4, 7, 8), but they are only present on median and posterior chaetigers, whereas in *M.setiuense* sp. nov., the pectinate chaetae are present throughout the body. The maximum number of branchial filament in *M.setiuense* sp. nov. (L10: 2.7 (2.85–4.8) mm) is five, and up to seven for *M.iloiloensis*. *Marphysamultipectinata* (L10: 13.9 mm) has palmate branchiae with maximum five branchial filaments and begin from chaetiger 32 whereas *Marphysasetiuense* sp. nov. also has a maximum of five branchial filaments but they begin from chaetiger 20 (15–25). *Marphysasetiuense* sp. nov. and *M.multipectinata* have unidentate and bidentate subacicular hooks from chaetiger 25 (21–38) and chaetiger 20, whereas *M.iloiloensis* has unidentate subacicular hooks only from chaetiger 30–38. All these species have different formulae for MII, MIII, and MIV (see Table 6).

The other two *Marphysa* species of the Sanguinea complex occurring within the South China Sea, *M.tribranchiata* and *M.tripectinata* differ from *M.setiuense* sp. nov. by having no eyes. They also can be differentiated by the number of types of pectinate chaetae. *Marphysatribranchiata* and *M.tripectinata* have three types of pectinate chaetae, while *M.setiuense* sp. nov. has four (types 1, 2, 7, 8). *Marphysatribranchiata* lacks thin, wide isodont (type 2), while *M.tripectinata* lacks thin, narrow isodont pectinate chaetae (type 1). Also, *M.tripectinata* (L10: 12.7 mm) only has unidentate subacicular hooks, whereas *M.tribranchiata* (L10: 8.7 mm) and *M.setiuense* sp. nov. (L10: 2.7 (2.85–4.8) mm) have both unidentate and bidentate subacicular hooks.

*Marphysasetiuense* sp. nov. and *M.orientalis* differ by the presence or absence of eyes, shape of subacicular hooks, pair of anal cirri, the chaetiger on which the branchiae begin and the maximum number of branchial filaments. *Marphysasetiuense* sp. nov. has a pair of eyes and two pairs of anal cirri, while *M.orientalis* has no eyes and only one pair of anal cirri. The new species has unidentate and bidentate subacicular hooks while *M.orientalis* has only unidentate subacicular hooks. Branchiae in *M.setiuense* sp. nov. begin from chaetiger 20 (15–25) whereas in *M.orientalis* they occur from chaetiger 45. The maximum number of branchial filaments in *M.setiuense* sp. nov. is five, while *M.orientalis* only has three branchial filaments.

#### ﻿ Marphysa ibaiensis sp. nov.

Taxon classificationAnimaliaEunicidaEunicidae

02B523B8-A842-57E9-9251-CBE50DFBBA48

https://zoobank.org/EFD572B1-A215-49FF-9C42-76DA2561E4D9

**Figure 14.**
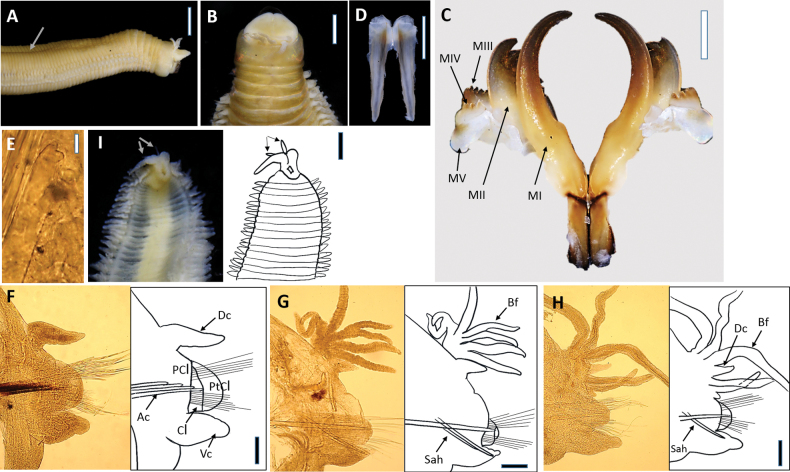
*Marphysaibaiensis* sp. nov. Holotype UMTAnn 2179 (**A–I**). Light microscopy images and digital drawing **A** anterior end, lateral view. Arrow shows shallow groove **B** anterior end, dorsal view **C** maxillae, dorsal view **D** mandibles, dorsal view **E** bidentate hook, chaetiger 97 **F** parapodium, chaetiger 10 **G** parapodium, chaetiger 97 **H** parapodium chaetiger 145 **I** posterior segments and pygidium, dorsal view. Arrows show the short pair of pygidial cirri. Abbreviations; MI–MV: maxillae I–V, Ac: aciculae, Dc: dorsal cirrus, Vc: ventral cirrus, PCl: prechaetal lobe, Cl: chaetal lobe, PtCl: postchaetal lobe, Sah: subacicular hook, Bf: branchial filament. Scale bars: 2 mm (**A, D**); 1 mm (**B, C, I**); 20 µm (**E**), 0.1 mm (**F–H**).

**Figure 15.**
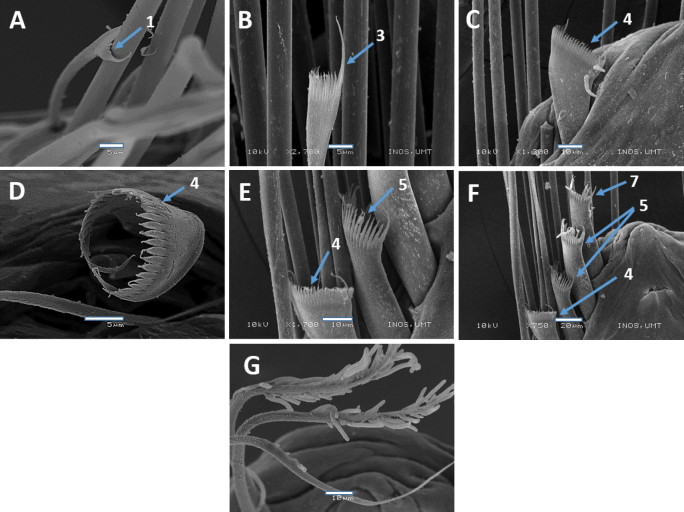
SEM images of *Marphysaibaiensis* sp. nov. Holotype UMTAnn 2179 (**B, C, E, F**), paratype AM W.54052 (**A, D, G**) **A** pectinate chaetae, chaetiger 97 **B** pectinate chaetae, chaetiger 10 **C** pectinate chaetae, chaetiger 88 **D** pectinate chaetae, chaetiger 88 **E–F** pectinate chaetae, chaetiger 125 **G** serrations and projections on limbate chaetae, chaetiger 31. Numbers denoted by arrows indicate the type of pectinate chaetae; 1. Thin, narrow isodont; 3.Thin, narrow heterodont; 4, 5. Thick, wide isodont; 7. Thick, wide anodont. Scale bars: 5 µm (**A–B, D**); 10 µm (**C, F–H**); 20 µm (**E**).

**Figure 16.**
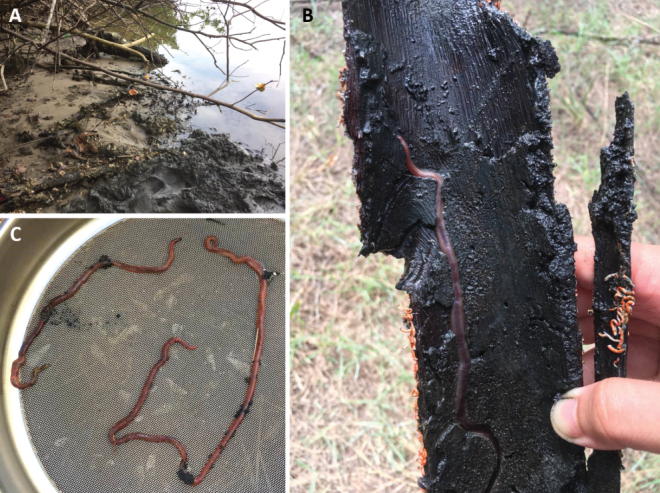
Sampling site in Kuala Ibai (estuary and lagoon area) **A** habitat of *Marphysaibaiensis***B** found in sediment deposited inside driftwood **C** live *M.ibaiensis* sp. nov.

Figs 1 , 2 , 14 , 15 , 16

##### Material examined.

***Holotype*.** UMTAnn 2179, complete, antero-ventrally dissected, some parapodia mounted for SEM. ***Paratypes*.**AM W.54052, complete, some parapodia mounted for SEM. LACM-AHF 13500 to 13502, complete, some parapodia removed; ZRC.ANN.1610 to 1612, complete, some parapodia removed; SAM-MB-A096022, complete, some parapodia removed. All material was collected from the east coast of Peninsular Malaysia, Terengganu, Kuala Ibai lagoon (05°17.198'N, 103°10.194'E) and estuary (05°16.780'N, 103°10.137'E), October 2021.

##### Diagnosis.

Prostomium completely bilobed, five prostomial appendages without articulations; eyes absent. Peristomium without Peristomial cirri. Maxillary apparatus with four pairs of maxillae, an unpaired on the left side, MI with falcal arch extended at sub-right angle, basal outer edge arched, basal inner edge lacking curvature. MII with triangular teeth and without attachment lamella. MIII slightly curved, with equal-sized triangular teeth, without attachment lamella. MIV with curved attachment lamella. Branchiae distributed along entire body. Dorsal cirri without articulations; postchaetal lobe well developed in anterior regions. Ventral cirri with swollen, inflated base. Sub-aciculae black, blunt, and translucent at distal end, pale brown in posterior-most parapodia. Supra-acicular chaetae include limbate, pectinate, thin, narrow isodont with short and slender inner teeth, pectinate thin, narrow heterodont with short and slender inner teeth, pectinate thick, wide isodont with long or short and slender inner teeth, and pectinate thick, wide anodont with long and slender inner teeth. Subacicular chaetae include limbate and compound spinigers. Subacicular hook bidentate. Pygidium with two pairs of anal cirri, without articulation.

##### Description

**(based on holotype, with variation in parentheses for paratypes).** Preserved specimens beige (Fig. 14A), ~ 195 (66–401) chaetigers and 52 mm (20–91 mm) long, L10: 4.5 mm (2.25–6.3 mm), W10: 2.85 mm (1.2–3.75 mm), excluding parapodia. Anterior region of body cylindrical, with shallow groove until median chaetigers (Fig. 14A); body depressed from chaetiger 30, elongated, and tapering at distal end. Live specimens red (Fig. 16C).

Prostomium conically bilobed, with two dorsoventrally lobes separated by an anterior notch (Fig. 14A, B). Prostomial appendages in a semicircle, median antennae separated by a gap. Palps, lateral and median antennae reaching first peristomium. Palpophores and ceratophores ring-shaped, short, and thin; palpostyles and ceratostyles tapering and slender. Prostomial appendage peduncles absent. Peristomium wider than prostomium; first ring 3× longer than second ring, separation between rings distinct on all sides.

Maxillae pale brown (Fig. 14C) and maxillary formula as follows: MF = 1+1, 6 (5–6)+7 (6–7), 7 (7–8)+0, 4+10 (9–10), 1+1. Maxillary carrier ~ 2.2× shorter than MI, rectangular anteriorly, triangular posteriorly. MI forceps-like, without attachment lamellae, falcal arch extended at sub-right angle, basal outer edge arched, basal inner edge lacking a curvature. Closing system is ~ 5.5× shorter than MI. Ligament between MI and MII pale brown. MII without attachment lamella, teeth triangular, present on < 1/2 of plate length. Ligament between MII and MIII pale brown. MIII single, longer than left MIV, slightly curved, with equal-sized triangular teeth, without attachment lamella. Left MIV short (< 1/2 the size of right MIV) with curved attachment lamellae. Right MIV long, with teeth triangular and curved attachment lamellae, decreasing in size and teeth curved posteriorly. MV paired. Mandibles whitish with pale brown core, longer than MI; cutting longer than MI; cutting plates whitish (Fig. 14D).

First few parapodia inserted ventrolaterally, but then becoming lateral in anterior region and dorsolaterally in subsequent segments. Chaetal lobes rounded on all chaetigers (Fig. 14F–H). Prechaetal lobe shorter than chaetal lobe along the entire body. Postchaetal lobe rounded and longer than chaetal lobe in anterior chaetigers and mid-body onwards (Fig. 14F–H), becoming shorter and absent in the posterior-most chaetigers. Dorsal cirri digitiform and slender, longer than ventral cirri anteriorly, as long as or shorter from mid-body and shorter in posterior chaetigers (Fig. 14F–H). Ventral cirri digitiform in first chaetigers, basally inflated with digitiform tip from chaetiger six onwards (Fig. 14F–H). Branchiae pectinate, starting from chaetiger 20 (11–65) and continuing to near end (~ 13 last chaetigers without branchiae), branchial filament 3× longer than dorsal cirri where best developed; number of filaments increasing from one anteriorly to eight in mid-body, decreasing to six in last several chaetigers. Pygidial cirri attached to ventral side of pygidium, dorsal pair ~ 4× longer than ventral (Fig. 14I).

Notoaciculae absent. Neuroaciculae black, blunt, and translucent at distal end along most of body, pale brown in posterior-most parapodia; ~ 3 or 4 per parapodium in anterior, one or two per parapodium in median and one per parapodium in posterior chaetigers (Fig. 14F–H). Supra-acicular chaetae with limbate capillaries and pectinates. Five types of pectinate chaetae were identified (types 1, 3, 4, 5, 7) (see Fig. 2): type 1: thin, narrow isodont with 12–15 short and slender inner teeth, present in anterior and median region (Fig. 15A); type 3: thin, narrow heterodont with 12 short and slender inner teeth, outer teeth longer on one side, present in the anterior body region (Fig. 15B); type 4: thick, wide isodont with 18–29 short and slender teeth, outer teeth different length to inner teeth, only present in median and posterior region (Fig. 15C–F): type 5: thick, wide isodont with 15–18 long and slender inner teeth, present only posteriorly (Fig. 15E, F); type 7: thick, wide anodont with ~ 15 long and slender inner teeth, only present in posterior parapodia (Fig. 15F). Subacicular chaetae with compound spinigers and limbate capillaries in median and posterior chaetigers. Some limbate chaetae with inconspicuous serrations and numerous projections (Fig. 15G). Subacicular hooks pale brown, translucent at distal end, from chaetiger 22 (22–46), 1–3 per parapodium; subacicular hooks bidentate present throughout (Fig. 14E). Pygidium with crenulated margin, with two pairs of tapering pygidial cirri attached to ventral side of pygidium, dorsal pair ~ 4× longer than ventral one (Fig. 14I).

##### Etymology.

Name refers to the type locality Kuala Ibai River.

##### Type locality.

South China Sea, Malaysia, east coast of Peninsular, Terengganu, Kuala Ibai river estuary and lagoon (see Fig. 1).

##### Distribution.

Known only from the type locality.

##### Habitat.

Slightly gravelly sand sediment (Table 4) associated with oyster clumps within *Rhizophora* spp. (Fig. 16A), burrowing in sediment deposited inside driftwood bark (Fig. 16B) with salinity 26‰ (estuary) and 18‰ (lagoon) during spring low tide.

##### Remarks.

With the presence of subacicular limbate and compound spinigers in the median and posterior region, *M.ibaiensis* sp. nov. belongs to Group E (Gravelyi). There are four *Marphysa* species belonging to this group; *M.borradailei* Pillai, 1958 (type locality: Negombo Lagoon, Sri Lanka), *M.fauchaldi* Glasby & Hutchings, 2010 (type locality: Ardatek Barrumundi farm, Darwin, Australia), *M.gravelyi* Southern, 1921 (type locality: Chilka Lake, India) and *M.madrasi* Hutchings, Lavesque, Priscilla, Daffe, Malathi & Glasby, 2020 (type locality: Chennai, India). The morphological features of these species are given in Table 7.

**Table 7.** Morphological features comparison between *Marphysa* Group E (Gravelyi) described in this study and species occurring worldwide. The features for new species are based on the holotype, with variation in parentheses for paratypes. Abbreviations: MF: maxillary formula, roman numerals refer to number of maxilla; PR-I: first peristomial ring; PR-II: second peristomial ring; p/a: present/absent; NIA: no information available. The major differences between the species are marked with asterisk (*).

Morphological feature	*M.borradailei* Pillai, 1958	*M.gravelyi* Southern, 1921	*M.fauchaldi* Glasby & Hutchings, 2010	*M.madrasi* Hutchings, Lavesque, Priscilla, Daffe, Malathi & Glasby, 2020	*M.ibaiensis* sp. nov.
Source of Information	Lectotype BMNH 1960.3.13.6 (Glasby and Hutchings 2010)	Paratypes 1938.5.7.55 and the type description (Hutchings et al. 2020)	Holotype NTM W23040 (Glasby and Hutchings 2010)	Holotype NL-ENNORE_01 (ZSI); Paratypes: ZSI-HQ/GNC/AN6072/1 (Hutchings et al. 2020)	Holotype: UMTAnn 2179 (this study)
Size (mm): L10, W10	NIA	NIA	NIA	6 (4–9), 2.5 (2–3.9)	4.5 (2.25–6.3), 2.85 (1.2–3.75)
Prostomium: shape	Bilobed	Bilobed	Bilobed	Bilobed	Bilobed
Palps: reaching	NIA	NIA	NIA	NIA	PR-I
Lateral antennae:reaching	NIA	PR-I	NIA	NIA	PR-I
Median antennae: reaching	NIA	PR-I	NIA	NIA	PR-I
Peduncle in prostomial appendages	NIA	Absent	Present	Absent	Absent
Eyes*	NIA	Present	Absent	Present	Absent
MF: MII, MIII, MIV*	6, NIA	5+6, 12–13+0, 4+8	5+6, 7+0, 4+9	8+9, 10+0, 7+11	6 (5–6)+7 (6–7), 7 (7–8)+0, 4+10 (9–10)
Branchiae: shape		Pectinate	Pectinate	Pectinate	Pectinate
Branchiae: start chaetiger; last chaetiger before pygidium	7–60; end ~ 10 last chaetiger	22–52; end ~ 20 last chaetiger	31; end ~ 10 last chaetiger	48–50, end ~ 10 last chaetiger	20 (11–65); end ~ 13 last chaetiger
Branchial filaments: numbers	20	9	9	9	8
Dorsal cirri: shaped	NIA	NIA	Conical	Digitiform	Digitiform
Prechaetal lobe: shaped	NIA	NIA	Ridge	Transverse fold	Transverse fold
Chaetal lobe: shaped	NIA	NIA	NIA	Rounded, conical	Rounded
Aciculae: shape; colour	NIA; black	NIA; black	NIA; oblique	Blunt, black with paler tips	Blunt; black and translucent at distal end
Subacicular limbate chaetae: (p/a); distribution	Present; NIA	Present; all chaetigers	Present; posterior chaetigers	Present; all chaetigers	Present; in median and posterior chaetigers
Pectinate chaetae: number of type*	NIA	NIA	2	2	5
Subacicular hook: shape; colour*	Unidentate, strongly hooded; NIA	Bidentate; NIA	Bidentate, close-fitting hood; dark brown	Bidentate; NIA	Bidentate; light brown and translucent at distal end
Subacicular hook: start chaetiger*	50	26–35	40	33–72	22 (22–46)
Subacicular hook: distribution	Continuous	Continuous	Continuous	Continuous	Continuous

*Marphysaibaiensis* sp. nov. can be distinguished from *M.borradailei* by the number of branchial filaments, shape of the subacicular hooks, chaetiger where the branchiae and subacicular hook occur, and the shape of postchaetal lobe in the anterior region. *Marphysaibaiensis* sp. nov. (TL: 52 (20–91) mm) has a maximum of eight branchial filaments whereas *M.borradailei* (TL: 1–8 mm) has up to 20 branchial filaments. The subacicular hook of *M.ibaiensis* sp. nov. is bidentate and occurs from chaetiger 22 (22–46) onwards while *M.borradailei* has a strongly hooded unidentate hook that occur from chaetiger 50 onwards. *Marphysaibaiensis* sp. nov. has rounded postchaetal lobe in anterior region, while *M.borradailei* has sub-conical shaped postchaetal lobes in the anterior region. The original description of *M.borradailei* makes it challenging to undertake a detailed morphological comparison and additional material from the type locality (Sri Lanka) needs to be collected and redescribed.

The new species can also be differentiated from *M.gravelyi* and *M.madrasi* by the absence of eyes, number of types of pectinate chaetae, number of branchial filaments, chaetiger where subacicular hooks begin and the length of the pygidial cirri. *Marphysaibaiensis* sp. nov. has no eyes, while both *M.gravelyi* and *M.madrasi* have a pair of eyes. *Marphysaibaiensis* sp. nov. has five types of pectinate chaetae (types 1, 3, 4, 5, 7), whereas *M.madrasi* has only two (types 4, 5). While all these species have bidentate hooks, they begin on chaetiger 22 (22–46) in *M.ibaiensis* sp. nov. (L10: 4.5 (2.25–6.3) mm), 26–35 in *M.gravelyi* and 33–72 in *M.madrasi* (L10: 6 (4–9) mm). *Marphysaibaiensis* sp. nov. has short and long pairs of pygidial cirri attached to the pygidium, whereas *M.madrasi* only has one pair of short pygidial cirri.

*Marphysaibaiensis* sp. nov. differs from *M.fauchaldi* by the absence of peduncle in prostomial appendages, the chaetiger on which the branchiae and subacicular hook occur and the distribution of subacicular limbate chaetae. Subacicular hooks and branchiae of *M.ibaiensis* sp. nov. (TL: 52 (20–91) mm) have a wide range variation of chaetiger where they begin; from chaetiger 22 (22–46) and 20 (11–65), respectively compared to *M.fauchaldi* (TL: 190 (78–155) mm); they begin from chaetiger 40 (31–50) and 31 (22–32), respectively. The subacicular limbate chaetae in *M.ibaiensis* sp. nov. occur from mid-chaetigers onwards whereas in *M.fauchaldi*, they are restricted to posterior chaetigers.

### ﻿Key to *Marphysa* species occurring in coastal water bodies of Malaysia and nearby areas (South China Sea and Andaman Sea)

**Table:** 

1	Compound chaetae present	**2**
–	Compound chaetae absent	**4**
2	Two types of compound chaetae present; spinigers and falcigers	***Marphysadigitibranchia* Hoagland, 1920**
–	One type of compound chaetae present; spinigers	**3**
3	One pair of anal cirri	***M.orientalis* Treadwell, 1936**
–	Two pairs of anal cirri	**5**
4	Subacicular hook absent	***M.kertehensis* sp. nov.**
–	Subacicular hook present	***M.moribidii* Idris, Hutchings & Arshad, 2014**
5	Subacicular limbate chaetae present	**6**
–	Subacicular limbate chaetae absent	**8**
6	Eyes absent, branchiae pectinate with ≤ 8 number of filaments	***M.ibaiensis* sp. nov.**
–	Eyes present, branchiae pectinate with ≤ 9 number of filaments	**7**
7	Subacicular hook bidentate and emerge from chaetiger 26–35	***M.gravelyi* Southern, 1921**
–	Subacicular hook bidentate and emerge from chaetiger 33–72	***M.madrasi* Hutchings, Lavesque, Priscilla, Daffe, Malathi & Glasby, 2020**
8	Branchiae palmate	***M.multipectinata* Liu, Hutchings & Sun, 2017**
–	Branchiae pectinate	**9**
9	Eyes present	**10**
–	Eyes absent	**12**
10	Subacicular hook unidentate and bidentate	***M.setiuense* sp. nov.**
–	Subacicular hook unidentate	**11**
11	Maximum number of branchial filaments seven, three types of pectinate chaetae	***M.iloiloensis* Glasby, Mandario, Burghardt, Kupriyanova, Gunton & Hutchings, 2019**
–	Maximum number of branchial filaments five, five types of pectinate chaetae	***M.merchangensis* sp. nov.**
12	Four types of pectinate chaetae	***M.hongkongensa* Wang, Zhang & Qiu, 2018**
–	Three types of pectinate chaetae	**13**
13	Maximum number of branchial filaments three	***M.tribranchiata* Liu, Hutchings & Sun, 2017**
–	Maximum number of branchial filaments eight	***M.tripectinata* Liu, Hutchings & Sun, 2017**

## ﻿Discussion

Prior to this study, a total of ten *Marphysa* species were described from Malaysia and nearby coastal waters (South China Sea and Andaman Sea) including one species from Group A (Mossambica) – *Marphysamoribidii*, six species from Group B (Sanguinea) – *M.iloiloensis*, *M.hongkongensa*, *M.multipectinata*, *M.orientalis*, *M.tribranchiata*, and *M.tripectinata*, one species from Group D (Belli) – *M.digitibranchia* Hoagland, 1920 (type locality: Hong Kong), and two species from Group E (Gravelyi) – *M.madrasi* and *M.gravelyi*. This study increases the number of *Marphysa* species from these water regions to 14.

Characteristics such as the distribution of different types of chaetae, including pectinate chaetae, branchial distribution and number of filaments, and jaw formula, allowed us to describe four new species. These characters have also been used recently by Lavesque et al. (2020) and Martin et al. (2020) in their studies of species of *Marphysa*.

All these new species occur in slightly different types of habitats, but share several general characteristics: all are found in mangrove areas, tolerate a wide range of salinity (euryhaline), and live in high percentage of sand. According to Glasby and Hutchings (2010), habitat type is a useful character to recognise species in a particular area. Therefore, describing a species’ habitat is important for taxonomic studies and conservation strategy management.

Phylogenetic analysis from COI data placed *M.merchangensis* sp. nov. as sister to *M.hongkongensa*, *M.setiuense* sp. nov. as sister to *M.iloiloensis*, *M.ibaiensis* sp. nov. as sister to *M.madrasi*, and *M.kertehensis* sp. nov. as sister to *M.mossambica* (Peters, 1854). Nevertheless, the interspecific divergence between these new species and all their sister taxa pair is high (Pair-wise Kimura 2-parameter – COIK2P range 6.14%–19.16% (see Suppl. material 1), which clearly showed the distinct genetic separation. Additionally, obtaining sequence data for *M.moribidii* is imperative to investigate the genetic difference between *M.moribidii* and *M.kertehensis* sp. nov. as they possessed a few similar morphological features and occur within Malaysian water bodies. The molecular analysis in this study aligns with the morphological analysis and confirms the presence of four new *Marphysa* species in the Terengganu mangrove area.

## ﻿Conclusions

Four species of *Marphysa* from Terengganu mangrove forests (lagoon, river, and estuary) were described and confirmed by morphology and molecular data and can also be separated based on their habitat. This study increases the species in the genus *Marphysa* and the number of polychaetes described from Malaysia. In addition, data provided in this study can also provide insight for future research on the potential use of *Marphysa* species in Malaysia as the only described species in Malaysia, *M.moribidii* has revealed a wide potential application for commercial use.

## Supplementary Material

XML Treatment for Marphysa

XML Treatment for Marphysa kertehensis

XML Treatment for Marphysa merchangensis

XML Treatment for Marphysa setiuense

XML Treatment for Marphysa ibaiensis

## ﻿Additional information

### Author ORCIDs

Che Engku Siti Mariam Che Engku Abdullah https://orcid.org/0009-0002-8371-1141

Izwandy Idris https://orcid.org/0000-0003-1516-8175

Afiq Durrani Mohd Fahmi https://orcid.org/0000-0002-5131-0098

Beth Flaxman https://orcid.org/0000-0002-0329-9525

Pat Hutchings https://orcid.org/0000-0001-7521-3930

### Data availability

The data underpinning the analysis reported in this paper are deposited at GBIF, the Global Biodiversity Information Facility, and are available at https://www.gbif.org/dataset/f3e0c6b6-0bcc-4a3b-9ee6-1ca8d1098f7c.

## Supplementary materials

Supplementary material 1 Pair-wise patristic distances between pairs of sequences of *Marphysa* species Data type xlsx https://binary.pensoft.net/file/1062343This dataset is made available under the Open Database License (http://opendatacommons.org/licenses/odbl/1.0/). The Open Database License (ODbL) is a license agreement intended to allow users to freely share, modify, and use this Dataset while maintaining this same freedom for others, provided that the original source and author(s) are credited.Che Engku Siti Mariam Che Engku Abdullah, Izwandy Idris, Afiq Durrani Mohd Fahmi, Beth Flaxman, Pat Hutchings
